# HBV and HBsAg strongly reshape the phenotype, function, and metabolism of DCs according to patients’ clinical stage

**DOI:** 10.1097/HC9.0000000000000625

**Published:** 2025-01-29

**Authors:** Lucile Dumolard, Theophile Gerster, Florent Chuffart, Thomas Decaens, Marie-Noelle Hilleret, Sylvie Larrat, Philippe Saas, Evelyne Jouvin-Marche, David Durantel, Patrice N. Marche, Zuzana Macek Jilkova, Caroline Aspord

**Affiliations:** 1 University Grenoble Alpes, Inserm U 1209, CNRS UMR 5309, Institute for Advanced Biosciences, Grenoble, France; 2 Hepato-Gastroenterology and Digestive Oncology Department, CHU Grenoble Alpes, Grenoble, France; 3 Etablissement Français du Sang Auvergne-Rhone-Alpes, R&D Laboratory, Grenoble, France; 4 University Grenoble Alpes, Laboratoire de Virologie, CHU Grenoble Alpes, Grenoble, France; 5 INSERM, U1111, Centre International de Recherche en Infectiologie (CIRI), University of Lyon (UCBL1), CNRS UMR_5308, ENS de Lyon, Lyon, France

**Keywords:** antiviral immunity, cDC1s, cDC2s, chronic hepatitis B, CX3CL1, dendritic cells, HBsAg, HBV, immunometabolism, pDCs

## Abstract

**Background:**

Hepatitis B is a liver infection caused by HBV. Infected individuals who fail to control the viral infection develop chronic hepatitis B and are at risk of developing life-threatening liver diseases, such as cirrhosis or liver cancer. Dendritic cells (DCs) play important roles in the immune response against HBV but are functionally impaired in patients with chronic hepatitis B. The underlying mechanisms involved in HBV-induced DC dysfunctions remain to be elucidated.

**Methods:**

We explored DC modulations by HBV and HBsAg by exposing blood-derived cDC1s, cDC2s, and plasmacytoid DCs from healthy donors to HBV or HBsAg and stimulating them with toll-like receptor ligand. Their phenotypic and functional features, as well as their metabolic profile, were analyzed through multiparametric flow cytometry and multiplex assays and further explored on patients’ samples.

**Results:**

We found that HBV deeply reshaped the DC secretome in response to toll-like receptor ligand. Strikingly, we observed that HBV-exposed DCs secrete high levels of CX3CL1 (fractalkine), a chemokine responsible for attracting antiviral effectors to the site of infection. HBsAg exposure favored DC activation while drastically altering TRAIL expression in response to toll-like receptor ligand and increasing the secretion of cytokines/chemokines involved in immune tolerance. HBsAg further dampened the metabolism of DC subsets while driving metabolic switches. Notably, the relevance of the CX3CL1/CX3CR1 axis, TGF-β, and metabolic disturbances was demonstrated within intrahepatic DC subsets in patients according to disease stage.

**Conclusions:**

Our work brings new insights into the immunomodulation induced by HBV on DCs, which contribute to impaired antiviral responses and progression toward chronicity.

## INTRODUCTION

Whereas progression to chronicity often occurs upon infection in early life, HBV infection results in immune-mediated viral clearance in 90%–95% of adults while the remaining 5%–10% fail to control viral infection, leading to chronic hepatitis B. Currently, around 300 million people are chronically infected with HBV, despite the availability of an effective preventive vaccine. An estimated 15%–40% of patients who are HBV-infected may develop complications such as liver cirrhosis, liver failure, and HCC, which represent the common causes of HBV-related death.^1^ Nucleoside or nucleotide analogs (NUC) are currently the most frequently applied treatment for patients with chronic hepatitis B. Such antiviral drugs can efficiently inhibit viral replication but do not provide a complete cure, as the functional eradication of the virus is achieved in <10% of cases. The lack of curative treatment to eliminate the infection justifies the need to define new targets and develop new or combined therapeutic strategies.^2^ The immunological management of HBV infection is essential for the functional control of the virus. Indeed, patients resolving the infection successfully elicited cytotoxic T lymphocytes specific for viral antigens, specific antibodies, and functional natural killer (NK) cells allowing long-term control of the infection, whereas patients who progress to the chronic phase fail to mount an immune response sufficiently robust, functional, and sustained to clear the infection. The restoration of appropriate immune responses to the virus is, therefore, a promising therapeutic approach, but such achievement requires first deciphering HBV-associated immune-modulatory mechanisms that are not yet fully understood.

Dendritic cells (DCs) are key players in immunity, as they harbor a central role in the induction and regulation of immune responses. Due to their unique properties at the interface between innate and acquired immune responses, they play a crucial role in the induction and orientation of antiviral immunity. DCs can be classified into 3 main subsets: conventional DCs (cDCs) subdivided into type-1 BDCA3^+^ cDCs (cDC1s) and type-2 BDCA1^+^ cDCs (cDC2s), and plasmacytoid DCs (pDCs) expressing BDCA2.^3^ These DC subsets express different sets of pathogen recognition receptors including C-type lectin receptors (CLRs) or Toll-like receptors (TLRs). This allows them to recognize different molecular patterns expressed by pathogens, leading to the subsequent production of various proinflammatory (monocyte chemoattractant protein-1 [MCP-1], C-C motif chemokine ligand [CCL]3, CCL4, CCL5, TNF-α, CX3CL1, and IL-23), regulatory (thymus and activation regulated chemokine [TARC], macrophage-derived chemokine [MDC], IL-10, and TGF-β), or antiviral (IL-12p70, interferon [IFN]-α, IFN-β, IFN-λ1, and IFN-inducible protein [IP]-10) cytokines and chemokines. These factors inhibit viral infection while activating and recruiting innate and adaptive antiviral immune players. cDC1s secrete IFN-λs upon recognition of viral dsRNA by TLR3 and perform efficient cross-presentation of viral antigens from infected necrotic cells. cDC2s express mostly TLR4/8 and are the main producers of IL-12p70 through TLR8 signaling when interacting with single-stranded RNA.^3^,^4^ pDCs can recognize single-stranded RNA, as well as bacterial and viral DNA containing unmethylated CpG motifs through the expression of TLR7 and 9, respectively.^5^ pDCs exhibit a crucial role in antiviral immunity due to the early release of large amounts of type-I IFNs in response to viruses.^3^,^4^ DCs also upregulate costimulatory molecules (CD40, CD80, and CD86) and TRAIL during viral infections, allowing the direct killing of infected cells.^6^,^7^,^8^

Others and we demonstrated that the 3 subsets of DCs are phenotypically and functionally altered in the blood and liver of patients with chronic HBV, with decreased costimulatory molecule expression, modulation of immune checkpoint expression, altered CLR profile, and subsequent impaired cytokine production.^9^,^10^,^11^,^12^,^13^,^14^ We also highlighted an impaired maturation of circulating and hepatic pDCs and cDCs following stimulation with TLR agonists in patients with chronic HBV, as well as functionally altered pDCs, unable to trigger the cytotoxic activity of NK cells.^15^ Impaired immune responses are often associated with high circulating HBsAg levels,^16^,^17^ which suggests that HBsAg may be responsible for the immune system modulation. Even though the virus does not replicate in DCs, the presence of HBsAg within pDCs and cDC2s was already described.^14^,^18^,^19^ The role of HBsAg in immune subversion is reinforced by our recent observation showing that HBsAg inhibits DC function after TLR stimulation in a CLR-dependent and glycosylation-dependent manner.^10^

Besides, immunometabolism emerges as a critical factor in the regulation of immune cell functions.^20^ Recent studies identified the critical role of metabolic pathways, such as glycolysis, oxidative phosphorylation (OXPHOS), and fatty acid (FA) metabolism, in orchestrating DC function.^21^ Upon activation, cDCs increase their glucose metabolism by shifting from OXPHOS to glycolysis as the main energy source.^21^,^22^,^23^ Activated pDCs secrete massively type-I IFN, which is highly energy-consuming and requires rapid production of ATP. It was demonstrated that pDCs rather rely on the OXPHOS pathway to provide the energy supply for activation and production of type-I IFN through TLR7/9 ligation.^23^ Although HBV infection results in increased glycolysis in infected hepatocytes,^24^ it remains unknown to which extent the viral infection might hijack DC metabolism in chronic HBV diseases.

Altogether, DCs play a central role in launching effective antiviral responses, but HBV may exploit their plasticity to escape from immune control. To further explore the immune-modulatory properties of the virus, especially the mechanisms by which HBV may subvert DCs, we examined the impact of HBV, as well as HBsAg, on the features of the 3 major DC subsets (cDC1s, cDC2s, and pDCs) in terms of activation, function, and metabolic profile, and assess the relevance in patients. By identifying novel pathways of DC modulation by HBV, our work paves the way to develop innovative immunotherapeutic options reshaping proper antiviral immune responses and efficient control of the virus.

## METHODS

### Healthy donor and patient samples

This study protocol was approved by the ethics committee of Grenoble University Hospital (CHUGA), the “Comite de Protection des Personnes” (no. ID-RCB: 2018-A02164-51) and conformed to the French Blood Service’s (EFS-AuRA) Institutional Review Board (collection AC-2020-3959). Informed consent was obtained from all participants before their enrollment. Blood samples (dry tubes for serums and heparinized tubes for cells) were collected from patients who were HBV-infected (n = 70) and healthy donors (n = 26), respectively. Serums were collected upon centrifugation. Peripheral blood mononuclear cells were obtained by Ficoll density gradient centrifugation and stored at −150 °C until use. The clinical characteristics of the patients are summarized in Supplemental Table S1, http://links.lww.com/HC9/B870.

### Phenotypic analysis by flow cytometry

Upon culture with HBV/HBsAg +/− TLR-L, PanDCs were stained with fluorochrome-labeled anti-human CD11c-PerCP/Cy5.5, HLA-DR-APC-H7, CD86-AF700, TRAIL-BV421 (BD), BDCA1/CD1c-PE/Cy7, Lineage cocktail (CD3/CD14/CD16/CD19/CD20/CD56)-BV510, CD45-BV570 (BioLegend), BDCA2/CD303-APC, BDCA3/CD141-APC (MiltenyiBiotec), CD40-PE, and CD80-FITC (Beckman) antibodies. Dead cells were excluded with LIVE/DEAD-PE-TxRed cell stain (ThermoFisher). Stained cells were then fixed with FACS lysing solution and further analyzed using BD LSRII Flow Cytometer, FACSDiva (BD), and FCS Express-7 software. Among alive CD45^+^ cells, cDC1s were defined as Lin^–^HLA-DR^+^CD11c^int^BDCA3^high^ cells, cDC2s as Lin^–^HLA-DR^+^CD11c^high^CD1c/BDCA1^+^ cells, and pDCs as Lin^–^HLA-DR^+^CD11c^-^BDCA2^+^ cells.

## RESULTS

### Global understanding of how HBV and HBsAg affected DC functions

**FIGURE 1:**
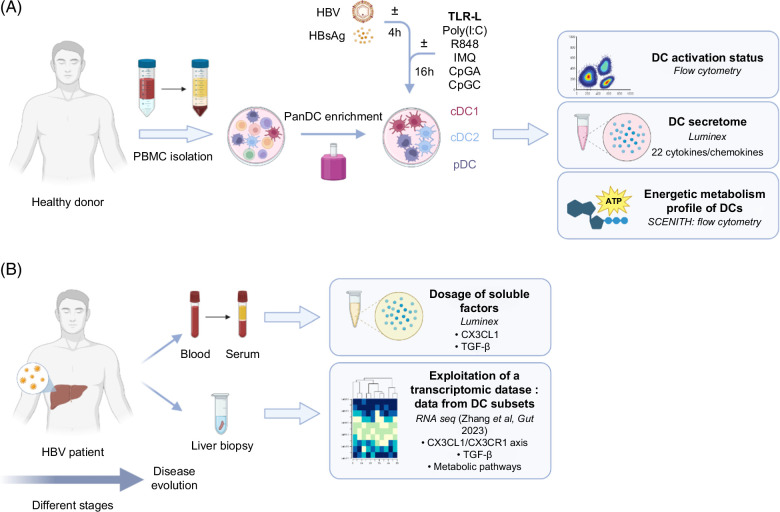
Experimental design. Schematic representation of the experimental layout used (A) to investigate the impact of HBV and HBsAg on DC activation, secretome, and energetic metabolic profile and (B) to assess relevance in patients. Created with BioRender. Abbreviations: cDC, conventional DC; DC, dendritic cell; PBMC, peripheral blood mononuclear cell.

To understand the mechanism of HBV escape from immunity, we explored whether HBV and HBsAg affect DC functions. PanDCs, containing a mixture of all 3 main DC subsets (cDC1s, cDC2s, and pDCs) were enriched from the blood of healthy donors and exposed to HBV or HBsAg for 20 hours in the presence or not of TLR ligands (TLR-L) targeting TLR3 (polyI:C), TLR7/8 (Resiquimod [R848] and imiquimod [IMQ]), and TLR9 (CpG_A_ and CpG_C_) (Figure 1). We examined both the direct impact of HBV/HBsAg on DCs and their influence on the ability of DC to respond to TLR stimulation. We depicted their activation status and TRAIL expression by flow cytometry, cytokine/chemokine secretion profiles by Luminex, and energetic metabolism using the SCENITH technology (Figure 1). A multiparametric flow cytometry strategy allowed the simultaneous analysis of the 3 major DC subsets (Supplemental Figure S1A, http://links.lww.com/HC9/B870). Such settings allow the depiction of direct impacts on individual DC subsets but also inter-DC cross-talks.

### HBV directly activated cDC2s and slightly affected the ability of DCs to upregulate the expression of activation molecules and TRAIL in response to TLR stimulation

**FIGURE 2:**
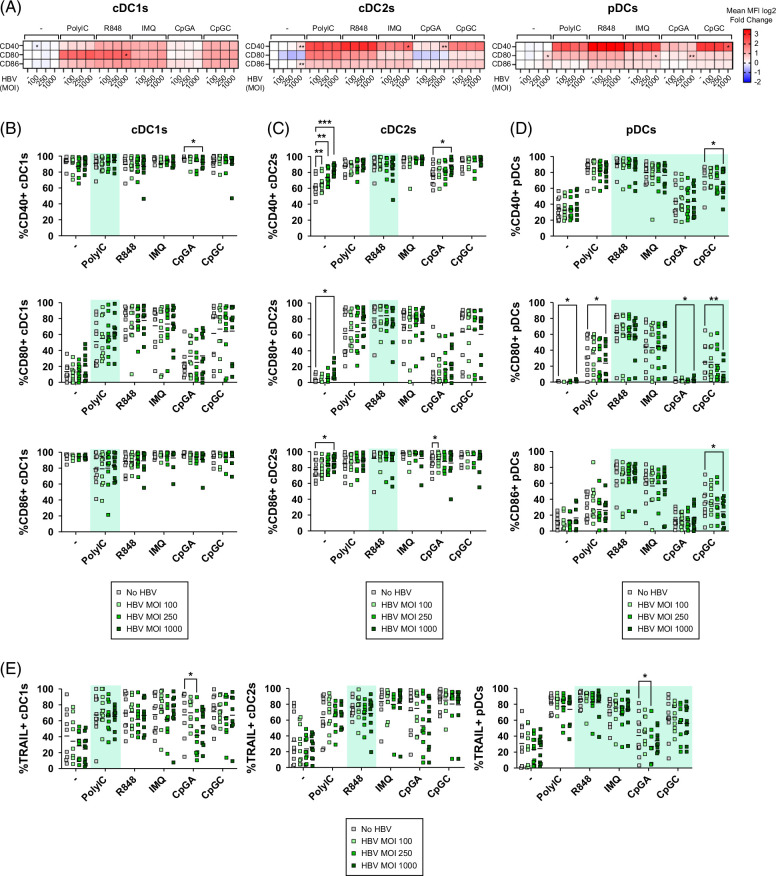
HBV directly activated cDC2s but slightly impacted the ability of DCs to upregulate activation molecules and TRAIL in response to TLR stimulation. PanDCs purified from HDs were cultured in the presence or not of HBV (MOI 100, 250, and 1000) for 4 hours and TLR3/7/8/9-L were then added for 16 hours. The subsequent modulation of activation markers (CD40, CD80, and CD86) and TRAIL on DC subsets was measured by flow cytometry. (A) Heat maps based on the fold change of the averaged MFI of surface markers expressed by cDC1s, cDC2s, and pDCs upon TLR stimulation after culture with or without HBV at different concentrations. Reference value (no stimulation [control], no virus [−]) settled as blank. Stars indicate significant differences within subgroups of stimulation compared to the condition without HBV (−). (B–D) Frequencies of CD40-, CD80-, and CD86-expressing cDC1s (B), cDC2s (C), and pDCs (D) upon TLR stimulation and after culture without (−) or with HBV at different MOI. (E) Frequencies of TRAIL-expressing cDC1s, cDC2s, and pDCs. Green colored background indicates TLR-L directly activating the DC subset expressing the corresponding TLR. Horizontal lines indicate means. *p* values were calculated using 2-way ANOVA with the Dunnett multiple comparison tests. *p < 0.05, **p < 0.01. n = 9–10 independent experiments. Abbreviations: cDC, conventional DC; DC, dendritic cell; HD, healthy donor; MFI, mean fluorescence intensity; MOI, multiplicity of infection; pDC, plasmacytoid DC; TLR, Toll-like receptor.

To analyze the impact of HBV on the activation status of DC subsets, PanDCs were incubated with HBV and further stimulated or not with TLR-L. We monitored the surface expression of costimulatory molecules CD40, CD80, and CD86, as well as TRAIL on DC subsets (Figure 2, Supplemental Figures S1B and 2, http://links.lww.com/HC9/B870). Heat maps based on the averaged fold changes in costimulatory molecule expression (MFI) by each DC subset compared to the control condition (no virus no stimulation) illustrated the direct impact of HBV on DCs (conditions “−”) together with the influence of HBV on the ability of DCs to respond to TLR stimulation (TLR-L conditions) (Figure 2A).

In the absence of TLR stimulation (condition “−”), HBV did not impact cDC1s (Figure 2B, Supplemental Figure S2A, http://links.lww.com/HC9/B870), whereas it increased the proportions of CD40^+^, CD80^+^, and CD86^+^ cDC2s and the expression levels of these markers (Figure 2C, Supplemental Figure 2B, http://links.lww.com/HC9/B870). HBV slightly increased CD80 and CD86 expression levels on pDCs (Figure 2D, Supplemental Figure 2C, http://links.lww.com/HC9/B870).

Under TLR stimulation, HBV did not affect the upregulation of costimulatory molecule expression by cDC1s and cDC2s following polyI:C and R848 stimulation, respectively (Figures 2B, C, Supplemental Figure S2A, B, http://links.lww.com/HC9/B870). However, HBV tended to enhance the proportions and/or expression levels of CD40 on cDC2s following CpG_A_ stimulation, probably through the cross-stimulation of pDCs (Figure 2C, Supplemental Figure S2B, http://links.lww.com/HC9/B870). Interestingly, HBV dampened the upregulation of CD40, CD80, and CD86 by pDCs triggered by CpG_C_ while slightly enhancing the intensity of CD80 upon IMQ and CpG_A_ stimulation (Figure 2D, Supplemental Figure S2C, http://links.lww.com/HC9/B870). Moreover, the upregulation of TRAIL expression induced by CpG_A_ stimulation was impaired by HBV on cDC1s and pDCs (Figure 2E). Altogether, these data show that HBV directly activates cDC2s and affects the capacity of pDCs to respond to TLR7/9 ligands. Importantly, HBV-induced immunomodulation of DCs is caused by factors present in the HBV inoculum, as UV-inactivated HBV drove a similar effect (Supplemental Figure S3, http://links.lww.com/HC9/B870).

### HBsAg directly triggered DC subset activation and altered the expression of activation markers and TRAIL on DCs in response to TLR stimulation

**FIGURE 3:**
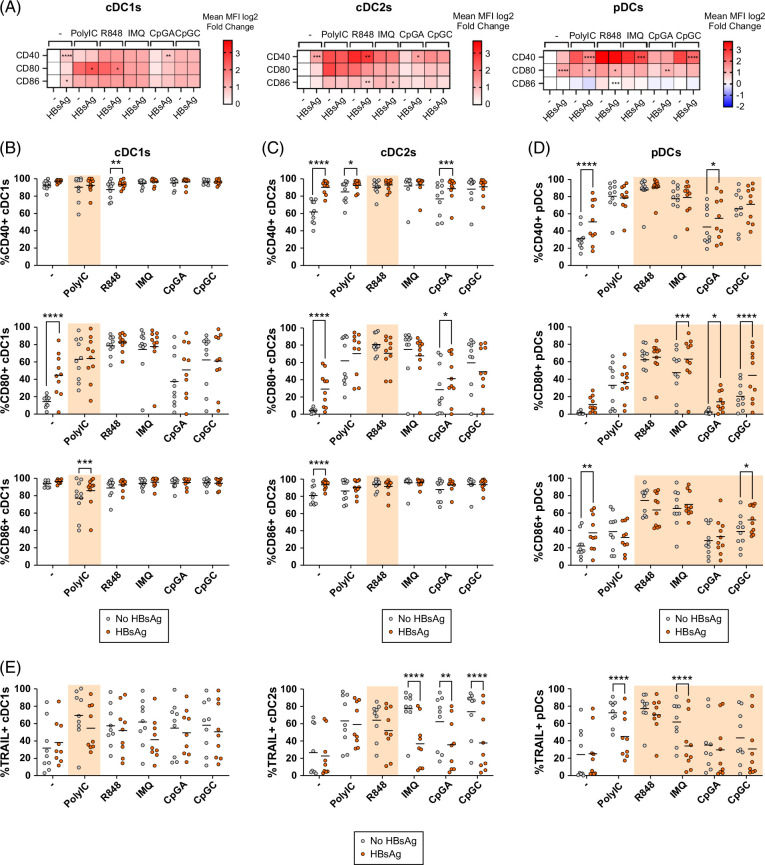
HBsAg directly triggered DC activation and altered the expression of activation markers and TRAIL on DCs in response to TLR stimulation. PanDCs purified from HDs were cultured in the presence or not of HBsAg for 4 hours, and TLR3/7/8/9-L were then added for 16 hours. The subsequent modulation of activation markers (CD40, CD80, and CD86) and TRAIL on DC subsets was measured by flow cytometry. (A) Heat maps based on the fold change of the averaged MFI of surface markers expressed by cDC1s, cDC2s, and pDCs upon TLR stimulation after culture without (−) or with HBsAg. Reference value (no stimulation [control], no HBsAg [−]) settled as blank. Stars indicate significant differences within subgroups of stimulation compared to the condition without HBV. (B–D) Frequencies of CD40-, CD80- and CD86-expressing cDC1s (B), cDC2s (C), and pDCs (D) upon TLR stimulation and after culture without (−) or with HBsAg. (E) Frequencies of TRAIL-expressing cDC1s, cDC2s, and pDCs. Orange colored background indicates TLR-L directly activating the DC subset expressing the corresponding TLR. Horizontal lines indicate means. *p* values were calculated using 2-way ANOVA with Šídák’s multiple comparisons tests. *p < 0.05, **p < 0.01, ***p < 0.001, ****p < 0.0001. n = 9–10 independent experiments. Abbreviations: cDC, conventional DC; DC, dendritic cell; HD, healthy donor; MFI, mean fluorescence intensity; pDC, plasmacytoid DC; TLR, Toll-like receptor.

Next, we studied the impact of HBsAg on the activation status of DC subsets. PanDCs were incubated with HBsAg and further stimulated or not with TLR-L. We monitored the surface expression of costimulatory molecules CD40/CD80/CD86, as well as TRAIL on DC subsets (Figure 3, Supplemental Figure S4, http://links.lww.com/HC9/B870). Heat maps based on the averaged fold changes in costimulatory molecule expression (MFI) by each DC subset were compared to the control condition (no virus no stimulation) illustrating the direct impact of HBsAg on DCs (conditions “−”) together with the influence of HBsAg on the ability of DCs to respond to TLR stimulation (TLR-L conditions) (Figure 3A).

In the absence of TLR stimulation (conditions “−”), HBsAg strongly activated all DC subsets, as illustrated by the increased frequencies of DCs expressing CD40, CD80, or CD86 (Figures 3B–D), and/or increased expression levels of these costimulatory molecules on DC subsets (Figure 3A, Supplemental Figure 4, http://links.lww.com/HC9/B870). This effect was dependent on HBsAg dose (Supplemental Figure S5, http://links.lww.com/HC9/B870).

Upon TLR stimulation, HBsAg enhanced the proportion of CD40^+^, CD80^+^, and/or CD86^+^-expressing cDC1s, cDC2s, and pDCs and/or intensity of expression of these markers upon TLR3 (polyI:C) and TLR9 (CpG_A_, CpG_C_) stimulation. In contrast, in the presence of TLR7/8-L (R848, IMQ), HBsAg dampened the expression level of activation markers for cDC2s and pDCs (Supplemental Figures S4B, C, http://links.lww.com/HC9/B870). Strikingly, HBsAg drastically impaired TRAIL expression by cDC2s and pDCs in response to TLR7 and TLR9 (IMQ and CpG_A/C_) stimulation, as well as TLR3 (polyI:C) for pDCs (Figure 3E). These results highlight that HBsAg directly triggers DC subset activation and alters their expression of activation markers and TRAIL in response to specific TLR stimulation.

Next, we used TBK1/IKK inhibitor Amlexanox (a TLR-signaling inhibitor) and Syk inhibitor R406 (a CLR-signaling inhibitor) to dissect the pathways involved in HBsAg and HBV’s direct activation of DCs and its effect on DCs’ responsiveness to TLR-L. Our results revealed the strong inhibitory capacity of TLR-signaling inhibitor Amlexanox on DC activation (Supplemental Figures S6 and S7, http://links.lww.com/HC9/B870), confirming the relevance of this signaling pathway for the HBV and HBsAg effect on the phenotype of DCs.

### HBV triggered the production of CX3CL1 and TGF-β by DCs and strongly modulated their secretome in response to TLR stimulation

**FIGURE 4:**
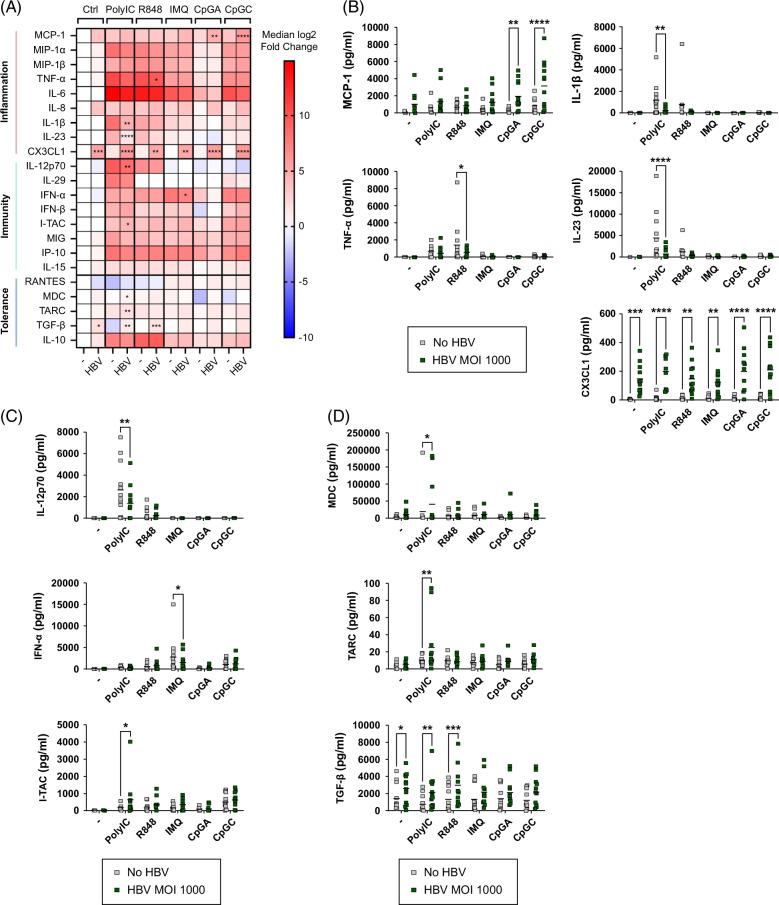
HBV triggered CX3CL1 production by DCs and modulated their secretome in response to TLR stimulation. PanDCs purified from HDs were cultured in the presence or not of HBV (MOI 1000) for 4 hours, and TLR3/7/8/9-L were then added for 16 hours. Secretion of cytokines/chemokines was measured in culture supernatants by Luminex. (A) Heat map based on the fold change of the median concentration of cytokines and chemokines secreted by PanDCs upon TLR stimulation after culture with or without HBV at an MOI 1000. Reference value (no stimulation [control, Ctrl] no virus [−]) settled as blank. Stars indicate significant differences within subgroups of stimulation compared to the condition without HBV (−). (B–D) Comparative concentration of selected cytokines and chemokines produced by DCs upon TLR stimulation after culture without (−) or with HBV and involved in inflammation (B), immunity (C), and tolerance (D). Horizontal lines indicate means. *p* values were calculated using 2-way ANOVA with Šídák’s multiple comparisons tests. *p < 0.05, **p < 0.01, ***p < 0.001, ****p < 0.0001. n = 10 independent experiments. Abbreviations: DC, dendritic cell; HD, healthy donor; MOI, multiplicity of infection; TLR, Toll-like receptor.

Cytokine and chemokine secretions by DCs are crucial to orientate the immune response toward a proinflammatory versus an anti-inflammatory response, and for the recruitment and activation of antiviral effectors. To decipher the impact of HBV on DC cytokine and chemokine secretion profiles, we cultured purified PanDCs in the presence, or not, of HBV and upon or not TLR triggering. We assessed in the culture supernatants a large panel of factors secreted by DCs involved in promoting inflammatory responses, triggering immune cell activation and functions, and inducing tolerance (Figure 4, Supplemental Figure S8, http://links.lww.com/HC9/B870). A heat map based on the median fold change of the analytes compared to the control condition (no virus, no stimulation) summarizes the modulations induced by HBV and the changes in the secretome of DCs upon TLR triggering (Figure 4A). Strikingly, HBV itself drives potent productions of CX3CL1 and TGF-β by DCs compared to DCs alone, independently of any TLR stimulation (Figures 4B, D). Importantly, CX3CL1 was not present in HBV suspension, and its secretion by DCs was increased whatever the TLR-ligand and induced by HBV in a dose-dependent manner (Supplemental Figure S9, http://links.lww.com/HC9/B870).

Interestingly, HBV dampened the production of antiviral cytokines (IL-12p70 and IFN-α) upon polyI:C and IMQ stimulation, respectively (Figure 4C). Production of regulatory mediators, such as MDC, TARC, and TGF-β, was increased following polyI:C stimulation (Figure 4D). In addition, HBV modulated proinflammatory factors, by enhancing production of MCP-1 upon TLR9 triggering and decreasing TNF-α, IL-1β, and IL-23 upon polyI:C or R848 stimulation (Figure 4B). Overall, HBV triggers CX3CL1 and TGF-β and reshapes the profile of cytokine/chemokine secretions of DCs in response to TLR stimulations.

### HBsAg induced the production of tolerogenic chemokines by DCs and reshaped the profiles of cytokine and chemokine secretions of DCs in response to TLR stimulation

**FIGURE 5:**
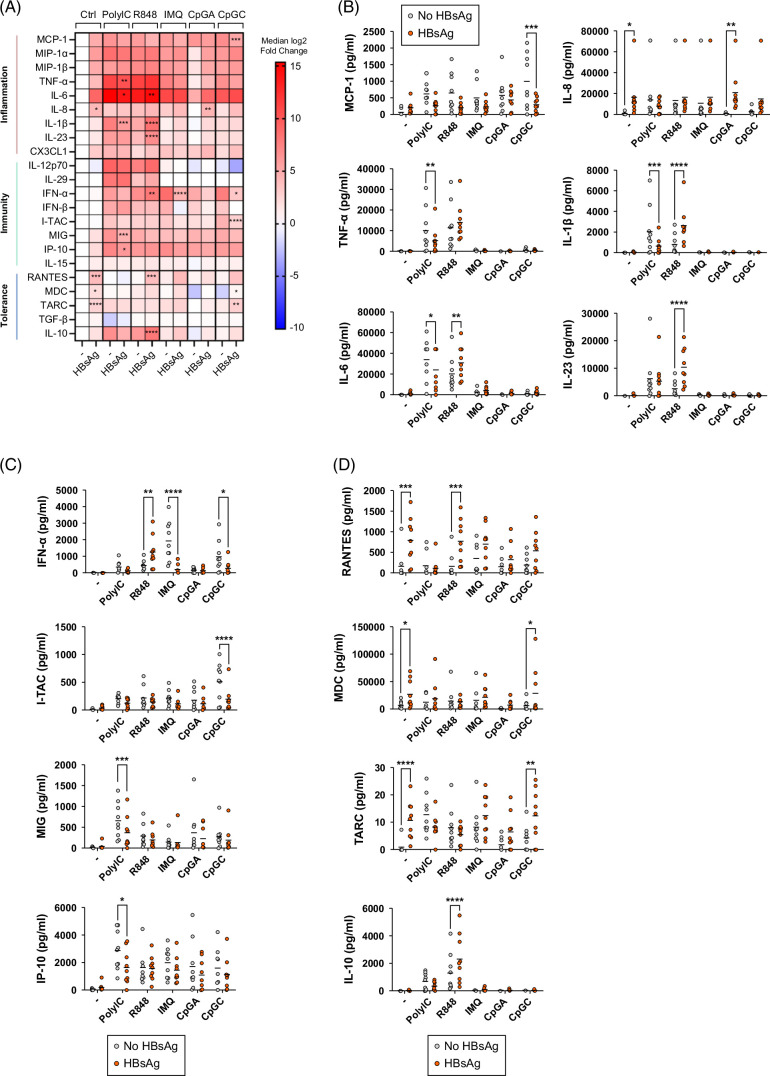
HBsAg reshaped cytokine and chemokine secretion profiles by DCs. PanDCs purified from HDs were cultured in the presence or not of HBsAg for 4 hours, and TLR3/7/8/9-L were then added for 16 hours. Secretion of cytokines/chemokines was measured in culture supernatants by Luminex. (A) Heat map based on the fold change of the median concentration of cytokines and chemokines secreted by PanDCs upon TLR stimulation after culture with or without HBsAg. Reference value (no stimulation [control, Ctrl] no virus [−]) settled as blank. Stars indicate significant differences within subgroups of stimulation compared to the condition without HBsAg (−). (B–D) Comparative concentration of selected cytokines and chemokines produced by DCs upon TLR stimulation after culture with or without HBsAg and involved in inflammation (B), immunity (C), and tolerance (D). Horizontal lines indicate means. *p* values were calculated using 2-way ANOVA with Šídák’s multiple comparisons tests. *p < 0.05, **p < 0.01, ***p < 0.001, ****p < 0.0001. n = 9 independent experiments. Abbreviations: DC, dendritic cell; HD, healthy donor; TLR, Toll-like receptor.

To investigate the influence of HBsAg on DC cytokine/chemokine secretion profiles, we cultured purified PanDCs in the presence or not of HBsAg and upon or not TLR triggering. We assessed in the DC culture supernatants the same 22 analytes analyzed in Figure 4 (Figure 5, Supplemental Figure S10, http://links.lww.com/HC9/B870). Heat map based on the median fold change of the analytes compared to the control condition (no HBsAg, no stimulation) outlines direct modulations induced by HBsAg and changes in the secretome of DCs upon TLR triggering (Figure 5A). HBsAg itself induced the production of proinflammatory cytokines and chemokines by DCs, although the effect was only significant for IL-8 (Figures 5A, B). Some regulatory chemokines (RANTES, MDC, and TARC) were highly secreted by DCs in the presence of HBsAg without any TLR stimulation (Figures 5A, D). Upon TLR triggering, HBsAg drastically reduced the secretion of antiviral mediators as observed for MIG and IP-10 upon polyI:C stimulation, IFN-α upon IMQ and CpG_C_ stimulation, and I-TAC following CpG_C_ stimulation (Figure 5C). Furthermore, HBsAg strongly enhanced the production of regulatory mediators such as RANTES and IL-10 upon R848 stimulation, and MDC and TARC upon CpG_C_ stimulation (Figure 5D). The effects of HBsAg on inflammatory factors were variable. HBsAg decreased MCP-1 production by DCs in response to CpG_C_ triggering (Figure 5B). While HBsAg also downregulated IL-6 and IL-1β secretions by DCs upon polyI:C stimulation, these cytokines, together with IL-23, were enhanced with R848 stimulation (Figure 5b). RNA-sequencing data confirmed the capacity of HBsAg to remodel DC transcriptome and significantly induce the expression of proinflammatory cytokine IL-8 and regulatory chemokine MDC, as well as the capacity to block the expression of chemoattractant MCP-1 (Supplemental Figure S11, http://links.lww.com/HC9/B870).

Together these data show that HBsAg induces tolerogenic chemokine production by DCs and reshapes their profiles of cytokine/chemokine secretions in response to TLR stimulation.

### HBV and HBsAg perturbed DC metabolism

**FIGURE 6:**
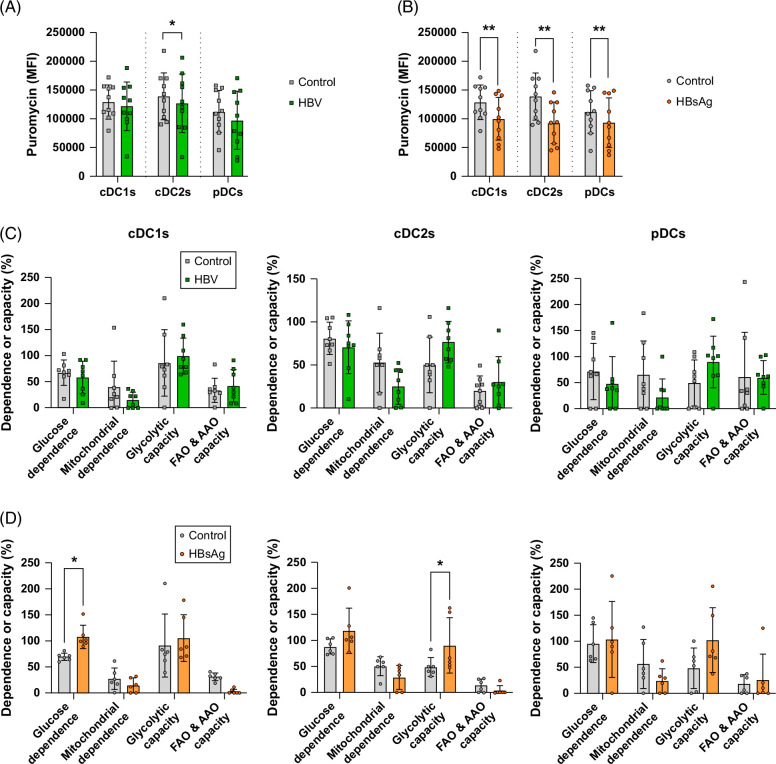
Global protein synthesis and metabolic profiles of DC subsets were perturbed by HBV and HBsAg. PanDCs purified from HDs were cultured in the presence or not of HBV/HBsAg for 16 hours without TLR stimulation, and incubated with the different inhibitors and puromycin following the SCENITH procedure. Translation levels were analyzed by measuring the MFI of puromycin within each subset using flow cytometry and metabolic profiles were calculated. (A, B) Translation levels within cDC1s, cDC2s, and pDCs with or without HBV (A) or HBsAg (B) exposure. Plots show mean with SD. n = 10 independent experiments. (C, D) Metabolic profiles of cDC1s, cDC2s, and pDCs with or without HBV (C) or HBsAg (D) exposure (n = 8 and 6 independent experiments, respectively). *p* values were calculated using Wilcoxon matched-pairs signed rank tests and 2-way ANOVA with Šídák’s multiple comparisons tests. *p < 0.05, **p < 0.01. Abbreviations: AAO, amino acids oxidation; cDC, conventional DC; DC, dendritic cell; FAO, fatty acids oxidation; HD, healthy donor; MFI, mean fluorescence intensity; pDC, plasmacytoid DC; TLR, Toll-like receptor.

To explore DC metabolism, we used the SCENITH (Single-Cell ENergetIc metabolism by profiling Translation inhibition) technology, which allows us to depict metabolism at a single-cell level by multiparametric flow cytometry.^25^ Protein synthesis (PS) reflects the global metabolic activity of a cell, mainly upon glycolysis and OXPHOS processes. SCENITH is based on the use of puromycin (puro); its incorporation is a reliable method for measuring PS level. Inhibition of the glycolysis and OXPHOS metabolic pathways by specific inhibitors (2-deoxy-glucose and oligomycin, respectively) leads to a drop in PS allowing a reliable determination of the metabolic pathways used. We found that HBV slightly reduced the global PS rate of cDC2s while having no effect on cDC1s and pDCs (Figure 6A). By contrast, HBsAg strongly reduced the translation levels of all DC subsets (Figure 6B). HBV did not significantly modulate DC metabolic profiles (Figure 6C). HBsAg induced a switch toward increased glucose dependency for cDC1s and glycolytic capacity of cDC2s (Figure 6D). Obviously, HBV and HBsAg induce perturbations of DC global metabolism. HBV decreased the global PS in cDC2s, while HBsAg affected more significantly DC metabolism with a reduction of PS in all 3 subsets and a shift toward glucose dependency for cDC1s and glycolytic capacity for cDC2s. We then evaluated whether CX3CL1 and/or TGF-β could be responsible for the metabolic deregulation of DCs by exposing PanDCs for 18 hours to rhCX3CL1 and/or TGF-β. cDC2s exhibited a reduced global PS upon incubation with TGF-β, without any significant alteration in their metabolic profile (Supplemental Figure S12, http://links.lww.com/HC9/B870).

### CX3CL1/CX3CR1 axis and TGF-β were major regulators during HBV infection in patients in both blood and liver compartments

**FIGURE 7:**
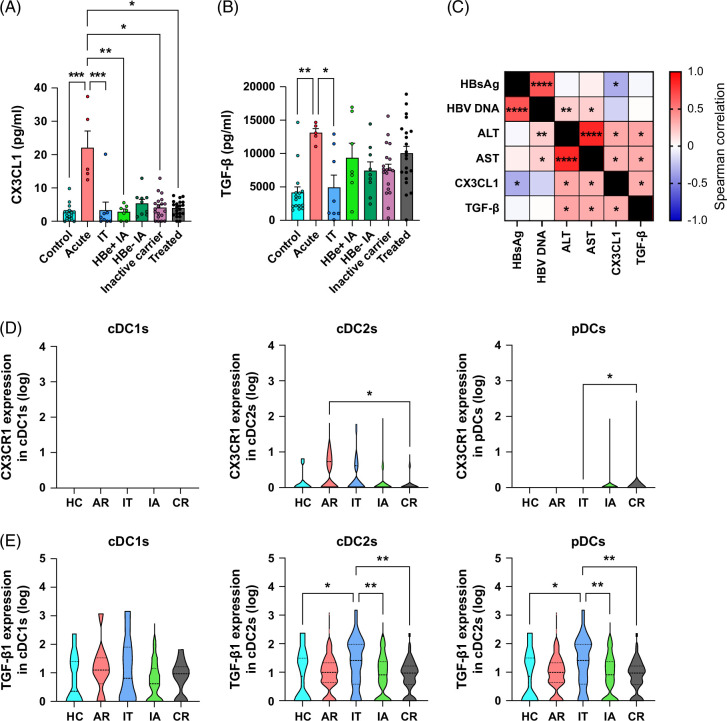
CX3CL1/CX3CR1 axis and TGF-β are major regulators during HBV infection in patients both in blood and liver compartments. (A–C) CX3CL1 (fractalkine) and TGF-β levels were analyzed in serums from patients with HBVat different stages of the disease and from healthy donors (control). (A) CX3CL1 level and (B) TGF-β level. *p* values were calculated using the Friedman test with the Dunn multiple comparisons tests. (C) Correlation matrix between circulating CX3CL1, TGF-β levels, and patients’ clinical parameters. Spearman correlation. (D, E) Expression of CX3CR1 (D) and TGF-β1 (E) transcripts within intrahepatic cDC1s, cDC2s, and pDCs of patients according to disease stage (exploitation of scRNAseq public data set GSE182159 from Zhang et al^26^). *p < 0.05, **p < 0.01, ***p < 0.001. Abbreviations: AR, acute recovery; CR, chronic resolved; HC, healthy control; IA, immune activation; IT, immune tolerant.

To assess the clinical relevancy of the previous findings, we analyzed in patients with HBV the presence of CX3CL1 and TGF-β both in circulation and within intrahepatic DCs at different stages of the disease. We found elevated levels of CX3CL1 and TGF-β in the serum of patients with acute HBV infection compared to control individuals or other disease stages (Figures 7A, B). Notably, CX3CL1 and TGF-β levels positively correlated between them, but also with ALT and AST levels, while CX3CL1 also negatively correlated with HBsAg level (Figure 7C). We then explored the CX3CL1/CX3CR1 axis as well as TGF-β1 transcripts within intrahepatic cDC1s, cDC2s, and pDCs of patients according to disease stage (Figures 7D, E) by exploiting the scRNAseq public data set GSE182159 from Zhang et al^26^. Although this data set lacks expression data for CX3CL1, we found a significant increase in CX3CR1 expression by intrahepatic cDC2s in patients undergoing acute recovery and by intrahepatic pDCs in chronic resolved patients (Figure 7D). In addition, TGF-β1 transcripts were significantly elevated within intrahepatic cDC2s and pDCs of patients in the immunotolerant phase compared to other disease stages or control conditions (Figure 7E). Expression levels of CX3CR1 and TGF-β1 were found in similar levels in whole intrahepatic CD45^+^ cells, supporting the crucial role of DC subsets in leading these axes in the context of HBV (Supplemental Figure S13, http://links.lww.com/HC9/B870). These findings highlight the role of the CX3CL1/CX3CR1 axis and TGF-β during HBV infection in patients in both blood and liver compartments.

### Glycolysis and OXPHOS metabolic pathways are disturbed within intrahepatic cDC1s and pDCs of patients according to the stage of the disease

**FIGURE 8:**
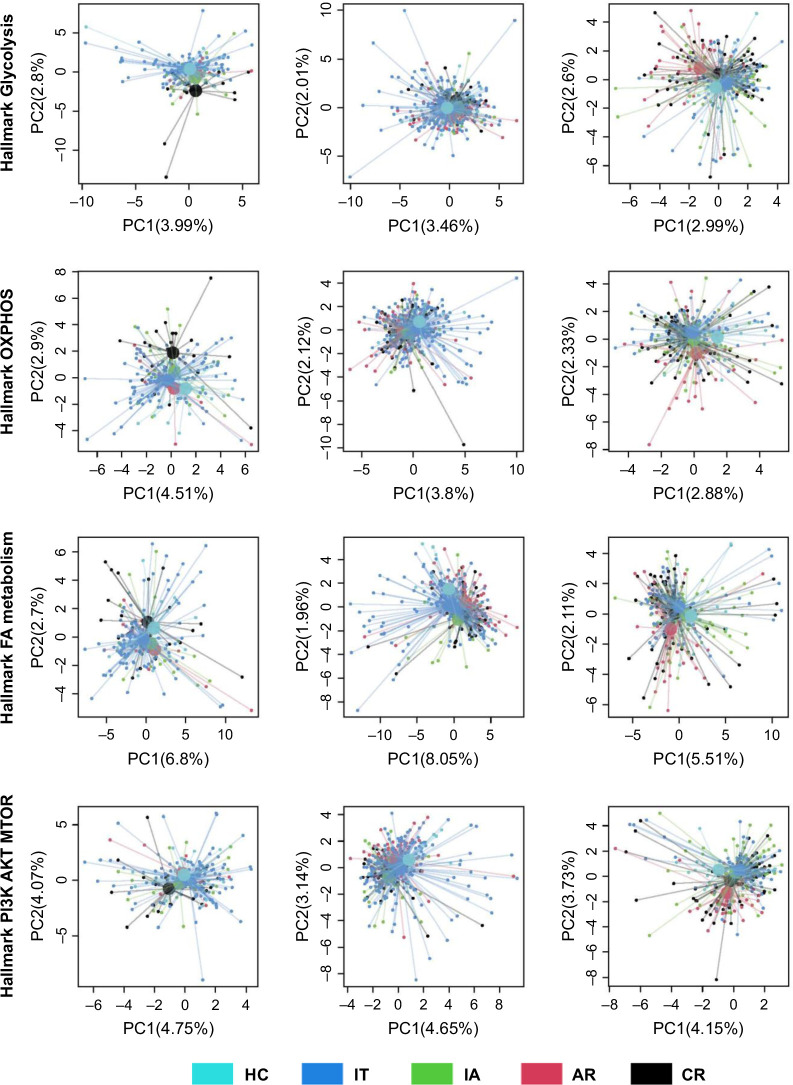
Disturbances of glycolysis and OXPHOS metabolic pathways within intrahepatic cDC1s and pDCs of patients according to the stage of the disease. Metabolic pathways (hallmark_glycolysis, hallmark_OXPHOS, and hallmark_FA metabolism) and their regulation (hallmark_PI3K AKT MTOR) were assessed within intrahepatic cDC1s, cDC2s, and pDCs of patients according to disease stage (exploitation of scRNAseq public data set GSE182159 from Zhang et al^26^). Plots displayed principal component projections 1 and 2 of the pathways. The colored circle represents the barycenter of the corresponding group. Abbreviations: AR, acute recovery; cDC, conventional DC; CR, chronic resolved; HC, healthy control; IA, immune activation; IT, immune tolerant; OXPHOS, oxidative phosphorylation; pDC, plasmacytoid DC.

We further explored in patients the status of the metabolic pathways (glycolysis, OXPHOS, and FA metabolism) as well as their regulation (known to involve PI3K/AKT/mTOR axes) within intrahepatic cDC1s, cDC2s, and pDCs according to disease stage by exploiting the scRNAseq public data set GSE182159 from Zhang et al^26^ combined to single-cell pathway analysis approaches (hallmark). We observed that glycolysis, OXPHOS, FA metabolism, and PI3K/AKT/mTOR pathways displayed distinct regulation within cDC1s for the chronic resolved group and within pDCs for the acute recovery group (Figure 8). Overall, these data demonstrate metabolic disturbances within intrahepatic cDC1s and pDCs of patients during the course of the disease.

## DISCUSSION

Understanding the immunomodulatory properties of HBV is crucial in achieving curative treatment. DCs display high functional plasticity, allowing them to orientate immunity toward multiple profiles depending on the microenvironment. This offers pathogens the opportunity to subvert DCs to escape from immune control. We previously reported major alterations in the functions of circulating and intrahepatic DC subsets in patients with chronic hepatitis B.^9^,^15^,^27^ To get further insights into the mechanism of such DC dysfunctions, we analyzed whether HBV and/or viral components could trigger such DC hijacking. Our study reveals that HBV and HBsAg affect many functions of DC subsets, including activation status, TRAIL expression, cytokine/chemokine secretion, and metabolism, and confirms major perturbations according to patients’ clinical stage (see Graphical abstract). Our findings bring new insights into the mechanism of subversion of DCs by HBV providing information on how the metabolic pathways interact with the immune response elements and contribute to viral persistence. This paves the way to develop new immunotherapeutic strategies to restore potent antiviral responses and efficient immune control of the virus.

First, we observed that HBV directly activated cDC2s, while HBsAg strongly activated all DC subsets. This is consistent with a previous study showing that exposure of blood cDC2s to HBsAg induced a strong DC maturation.^28^ However, previous studies reported that HBsAg lacked direct activating capacities on pDCs^13^ and cDC1s.^11^ Such discrepancies could result from the source of HBsAg. Indeed, interactions between HBsAg and DCs are glycosylation-dependent and occur through CLR.^10^ As glycosylation is species-specific, the source of HBsAg (yeast, mammalian cells, or patient blood) is important to adequately study in vitro interactions between HBV and DCs. In patients with chronic HBV, we demonstrated perturbations of the basal activation status of DC subsets,^9^ with a lower expression of CD80 on circulating cDC1s of patients with HBV, while intrahepatic cDC1s displayed higher CD40 expression, which correlated positively with HBsAg levels. This suggests that circulating cDC1s with an immature status become activated when localizing within HBV-infected livers. In addition, circulating and intrahepatic pDCs from patients displayed an upregulation of CD40 suggesting that HBV infection could also modulate pDC activation status.^9^,^10^,^27^ Altogether, these observations indicate that HBV and HBsAg can directly perturb DC activation in HBV patients.

Strikingly, we also reveal that HBV directly triggers major production of the proinflammatory chemokine CX3CL1 as well as the production of the immunosuppressive cytokine TGF-β1 by DCs, and the expression of these modulators in patients within intrahepatic DC subsets according to disease stage. This observation is consistent with reported studies in patients with HBV, showing upregulation of TGF-β1 associated with disease severity,^29^ and increased production of TGF-β1 by peripheral blood mononuclear cells.^9^ TGF-β1 stimulates the differentiation of Th17 cells, boosts the activities of CD4^+^ regulatory T cells, and contributes to liver disease progression.^30^ CX3CL1 is the main chemokine secreted by DCs upon contact with HBV. CX3CL1 is a chemoattractant for T cells, NK cells, and monocytes through CX3CR1.^31^ The secretion of CX3CL1 by DCs was never reported in HBV infection. Recent findings demonstrated by single-cell RNA-sequencing that the frequency of CX3CR1-expressing T cells can be used as a surrogate marker for viral resolution in patients with HBV.^26^ CX3CR1^+^ CD8^+^ central-memory T cells positively correlate with HBV clearance^26^ while the *CX3CR1* gene in T cells is downregulated in HBV-related HCC.^32^ CX3CL1 is also produced by hepatocytes and affects the migration activity of CX3CR1^+^ immune cells.^33^ Thus, the CX3CL1/CX3CR1 pathway may serve as an important regulator of immune responses in HBV infection, mainly by governing DC/effector crosstalk.

We further outline that HBV/HBsAg modulate the ability of DCs to respond to TLR stimulation. Whereas HBV slightly affected the ability of DCs to upregulate activation molecules and TRAIL in response to TLR stimulation, HBsAg strongly perturbed their expression of activation markers and reduced TRAIL. As TRAIL conferred direct cytotoxic potential to pDCs toward infected cells,^7^ its reduction could reflect a protection mechanism to avoid TRAIL-mediated killing of infected cells. Moreover, both HBV and HBsAg drastically reshaped the cytokine/chemokine secretion profiles of DCs in response to TLR stimulation. HBV dampened production of cytokines involved in antiviral immunity (IL12-p70 and IFN-α), while strongly enhancing regulatory mediators, such as MDC and TARC following TLR-L stimulation. In addition, HBV modulated proinflammatory factors, enhancing the production of MCP-1 upon TLR9 triggering while dampening the secretion of IL-1β, IL-23, and TNF-α upon TLR3/7/8 stimulation. HBsAg drastically reduced the secretion of antiviral mediators as observed for MIG, IP-10, IFN-α, and I-TAC upon TLR3/7/9 stimulation, while strongly enhancing the production of regulatory mediators such as IL-10, RANTES, TARC, and MDC upon R848 or CpG_C_ stimulation. HBsAg also influenced inflammatory factors, with decreased MCP-1 production by DCs upon CpG_C_ triggering. Altogether, these findings uncover a deep skewing of DC subsets by HBV that can reshape subsequent antiviral responses.

In the context of chronic HBV infection, functional impairments of DCs have been described,^11^,^18^,^34^,^35^ potentially through HBsAg.^28^ This is in line with our previous observations describing the inhibition of DC function in patients with HBV.^9^,^15^ Circulating cDCs and pDCs from patients with chronic HBV display an impaired antiviral cytokine production upon TLR triggering that correlates with viral parameters.^9^ Indeed, circulating cDC2s display a reduced ability to mature associated with a defective IL-12p70 and TNF-α production upon TLR triggering.^9^,^14^,^34^ Such cDC2 impairments correlated with plasmatic HBsAg and HBV DNA, indicating a direct impact of HBV and/or HBV proteins on cDC2 phenotype, maturation, and function.^9^ The effect of HBsAg on cDC2s purified from healthy donors was controversial, contributing to DC dysfunction^14^ or driving their strong activation in a TLR4-dependent and CD14-dependent manner^28^ depending on the microenvironment.

Regarding cDC1s, we and others previously demonstrated in patients with chronic HBV perturbations of their basal activation status, alteration of their maturation capacity, and deficient production of IFN-λ1, TNF-α, and IL-12 following TLR3 triggering.^9^,^11^ Correlations were found between cDC1 impairment and levels of HBsAg and HBV-DNA, suggesting a direct impact of HBV or HBV-antigens on cDC1 functions. cDC1s are prominently present in HBV-infected liver and enriched within hepatic DCs.^11^ cDC1s are the most potent producers of IFN-λ in response to viruses or synthetic RNA through TLR3 signaling^11^,^36^ and are specialized in antigen cross-presentation, therefore actively participating in the control of hepatotropic viruses.^11^ Few studies showed controversial impacts of IFN-λ on HBV replication in cell lines and in mouse models.^37^,^38^ However, in patients who are HBeAg-positive, PEG-IFN-λ was shown to induce a reduction of HBV replication,^39^ suggesting that this cytokine may be valuable in fighting chronic HBV infection.

For pDCs, we previously demonstrated that circulating and intrahepatic pDCs from patients with chronic HBV showed a defective maturation and a reduced IFN-α, and TNF-α production in response to TLR stimulation.^9^ Such functional impairment has been reported by other groups.^18^,^34^ We also revealed correlations between pDC modulations and levels of HBsAg and HBV-DNA, suggesting that the pDC status was strongly linked to viral parameters.^9^ Reduction of IFN-α secretion following TLR9 activation of circulating pDCs led to a defective triggering of NK cytotoxicity.^15^ Another study showed that HBV actively inhibits pDC function through HBsAg and HBeAg, potentially by BDCA2 binding.^13^

Our findings further highlight the potent cross-regulation between DC subsets. Indeed, we observed modulations in DC subsets that were not able to respond to the TLR-ligand, implying that modulations occurred through inter-DC cross-talk. pDCs through type-I IFN could provide critical licensing signals to cDCs,^40^ while IFN-λ1 potentiates IFN-α production.^41^ Therefore, HBV can influence DC subsets directly, and by exploiting inter-DC communication.

Taken together, HBV triggers functional defects in cDC2s, cDC1s, and pDCs that might contribute to the failure to properly elicit antiviral immune responses required for long-term viral control. Hence, HBV hijacks the immune system by subverting DCs for the benefit of the persistence of the viral infection.

Despite being a small cell cluster, DCs are crucial immune sentinels, which orchestrate antiviral immunity and display a crucial role in orientating antiviral responses and determining the outcome of infection. Yet HBV may exploit their plasticity to escape immunity: pDCs essential for type-I IFN response that is a strong innate immune defense mechanism and key in the antiviral program and modulation of adaptive immunity, BDCA3^+^ cDC1s crucial for cross-presentation and CD8 T-cell responses, and BDCA1^+^ cDC2s key for CD4 T-cell responses. Moreover, it has been demonstrated that increased infiltration of intrahepatic DC subsets closely correlates with viral control and liver injury.^42^ Thus, HBV hijacks the immune system by subverting DCs, which can then critically influence viral immune control or escape. Based on the crucial role played by DCs in orientating antiviral responses, DC-based immunotherapy represents a potent therapeutic option for chronic HBV infection.^43^

As DCs subsequently trigger the recruitment and activation of effector cells able to destroy infected cells through appropriate chemokines/cytokines, perturbations of DC secretome should influence the orientation of subsequent responses.^44^ Our work brings insights into the molecular framework explaining how HBV induces dysfunction in the DC/effector crosstalk. Major disturbances of DCs will affect subsequent crosstalk with antiviral effectors, and contribute to the failure to properly elicit antiviral immune responses required for long-term viral control. Phenotypic and functional alterations of DCs by HBV may subsequently impair proper cross-presentation of viral antigens,^45^ skew activation of cytotoxic effectors such as T and NK cells,^15^ and prevent elicitation of B-cell immunity,^46^ which is ultimately required to counteract HBV infection.

We highlight that HBV and HBsAg provoke changes in DC metabolism and metabolic switches between OXPHOS and glycolysis pathways, and demonstrate such metabolic perturbations within intrahepatic DC subsets. Recent studies identified the critical role of metabolic pathways, such as glycolysis, OXPHOS, and FA metabolism, in orchestrating DC functions.^21^,^22^ Distinct metabolic states guide the development of inflammatory versus tolerogenic DCs.^47^ Metabolic pathways strongly affect DC immune profiles. Following Influenza infection, DCs respond with a global metabolic reprogramming critical for effector function.^48^ Thus, metabolic switching and reprogramming in DCs is crucial to initiate a proper response to infection. By driving perturbations of DC metabolism, HBV may contribute to the subsequent failure of DCs to trigger efficient antiviral responses and drive subsequent immunity toward chronicity. Immunometabolism can be considered an interesting therapeutic target to strengthen the efficacy of antiviral and immunotherapies.

Thus, our findings bring novel insights into the mechanisms of HBV subversion of DCs and escape from immune control, which is critical to propose innovative immunotherapeutic options reshaping proper antiviral responses and efficient control of the virus. In combination with other therapeutic strategies,^2^,^49^ especially DC-based strategies^43^ developed for chronic HBV infection, or antiviral drugs, our work provides bases for designing innovative immunotherapeutic strategies aiming at restoring DC functions through unlocking of the inhibition triggered by the virus. This may allow the restoration of efficient immune control of the virus and prevent the chronicity of infection.

## Supplementary Material
