# Supplementary material for: HBV and HBsAg strongly reshape the phenotype, function, and metabolism of DCs according to patients’ clinical stage
Source: Hepatol Commun. 2025 Jan 29;9(2):e0625. doi: 10.1097/HC9.0000000000000625 (PMC11781764; doi:10.1097/HC9.0000000000000625)

Supplementary Material and Methods

Virus and reagents

HBV inocula were prepared by concentrating supernatant from HBV-infected hepatocyte cell line as described previously [1]. HBV particles were used at a multiplicity of infection (MOI) of 100, 250 or 1000vge/ml. As reported previously [1,2,3], 100 vge/mL of viral particles contains the equivalent of 5ng/mL of HBsAg. Purified HBsAg (adw subtype) from human HBsAg-positive serum (MyBioSource, MBS318792) was used at a concentration of 25µg/ml (e.g. 25 000 UI/mL). In control experiment, HBV virus was UV-inactivated through UV light exposure (16h). Synthetic TLR-L including polyinosinic-polycytidylic acid (polyI:C, 30µg/mL), Resiquimod (R848, 1µg/mL), Imiquimod (IMQ, 1µg/ml), Class-A CpG oligonucleotide ODN-2336 (CpG_A, 1µM) and Class-C oligonucleotide ODN-2395 (CpG_C, 1µM) were obtained from InvivoGen.

Dendritic cell isolation and culture

Culture medium was prepared with RPMI-1640/GlutaMAX (Invitrogen) supplemented with 1% MEM (100X) non-essential amino acids (Invitrogen), 20µg/mL Gentamycin (Invitrogen) and 1mM Sodium Pyruvate (Sigma-Aldrich)) with 10% human serum (HS). PanDCs (containing a mixture of cDC2s, cDC1s and pDCs) were enriched from thawed PBMCs by negative selection using the EasySep™ human Pan-DC pre-enrichment kit (StemCell) according to manufacturer's instructions. Purified panDCs were plated at 2.10^6 cells/ml in culture medium in a 96-well round-bottom plate and incubated with or without HBV at different MOI (100, 250 and 1000) or HBsAg (25µg/ml) at 37°C, 5% CO2. After 4h of incubation, TLR-L (including Poly(I:C), R848, IMQ, CpG_A or CpG_C) were eventually added, and cultures were performed for 16h. In some experiments, PanDCs were pre-treated for one hour with specific inhibitors of the TLR- and CLR-mediated pathways before adding virus or HBsAg: amlexanox (150µg/ml, Invivogen), inhibitor of the noncanonical IkB kinases TANK-binding kinase 1

(TBK1) and IkappaB kinase-epsilon (IKKε) (e.g. inhibitor of the interferon regulatory factor 3 (IRF3)- and NF-κB-mediated inflammatory signaling pathways), or R406 (2μM, Invivogen), inhibitor of the spleen tyrosine kinase (Syk) pathway, In some experiments, PanDCs were also cultured in the presence of rhCX3CL1 (25ng/mL) and rhTGF-β (5ng/mL) (StemCell).

Analysis of cytokine and chemokine secretion using Luminex

Patients' serum and supernatants from panDC cultures were harvested and interleukin (IL)-1β, IL-6, IL-8, IL-10, IL-12p70, IL-15, IL-23, IL-29, interferon (IFN)-α, IFN-β, transforming growth factor (TGF)-β (active form), tumor necrosis factor (TNF)-α, CX3CL1, IP-10, I-TAC, MCP-1, MDC, MIG, MIP-1α, MIP-1β, RANTES and TARC secretions were measured by Luminex™ technology using MAGPIX® 200™ Instrument with xPONENT® software (Bio-Rad) and analysed using ProcartaPlex Analysis App.

Analysis of cell metabolism using SCENITH

Purified PanDCs were seeded in 96-well plates at 2.10^6 cells/mL in culture medium in the presence or not of HBV (MOI 1000) or HBsAg (25µg/ml) for 16h. After washing and resuspension in RPMI 10% FCS, cells were treated during 30min with DMSO (Control), 2-Deoxy-D-Glucose (DG, 100mM, Sigma), Oligomycin (Oligo, 1mM, Ozyme), or a combination of these drugs. Puromycin (Puro, 10mg/mL, Cayla) was then added during 15min and cells washed in cold PBS. Then, staining with fluorescent cell viability marker and primary conjugated antibodies against surface markers were performed during 20min at 4°C in PBS. After washing, cells were fixed and permeabilized using FOXP3 fixation and permeabilization buffer (Thermofisher) following manufacturer instructions. Intracellular staining of puromycin was performed by incubating cells during 30min at 4°C with anti-Puro monoclonal antibody (Merck). Stained cells were then washed and fixed with FACS lysing solution (BD) and further analysed using BD® LSRII Flow Cytometer, BD FACSDiva™ and FCS Express-7 softwares (BD). Metabolic profiles were calculated as described previously [25]. Experiments showing

abnormal percentage for dependency and capacity were excluded for the determination of metabolic profiles (>250%).

RNAseq analyses of HBsAg-subverted FACS-sorted PanDCs

PanDCs were first enriched from PBMC derived from HDs using the EasySep PanDC pre-enrichment Kit (StemCell), further labelled with BV421 anti-CD11c, PerCP anti-HLA-DR and BV510 anti-Lin, and submitted to FACS sorting (BD Aria) to isolate pure PanDCs (Lin^{-} HLA-DR^{+}) (purity >99,8%). FACS-sorted PanDCs were then cultured for 20 hours with HBsAg (25µg/mL). Cells were then washed in PBS, and pellets resuspended in RNAlater for subsequent RNAseq analyses. The total RNA was extracted from cells (n=4/group) preserved in an RNA stabilization solution. The RNA purification was performed with RNeasy Mini Kit® (Qiagen, Hilden, Germany), according to the instructions provided by the manufacturer and quality assessment for the RNA Integrity Number (RIN) was performed using Agilent 2100 Bioanalyzer (Agilent, Palo Alto, CA, USA). The libraries was prepared using the SMARTseq2 method-based mRNA library construction, according to the manufacturer protocol. A total of 16367 genes were detected. The normalization and differential analysis were performed using the Dr Tom software.

Single-cell RNA sequencing (scRNAseq) data processing

We exploited the scRNAseq public dataset GSE182159 from Zhang *et al.*[4]. Due to the patient privacy restrictions and the China's Human Genetic Resources Policy, the raw data files have not been provided. We used preprocessed (as described in [4]) data, available on the NCBI/GEO data warehouse. We focus on the 106592 liver cells from 23 patients. We first performed hierarchical clustering of 106592 cells according to scRNAseq signal of the 60 markers to identify DC subsets (cDC1s, cDC2s, pDCs) based on markers defined in [4] (fig. S12-A). We selected cells that specifically express markers of cDC1s, cDC2s and pDCs. We then assessed in cell subsets and across disease stages (6 immune tolerant, 5 immune active, 3 acute resolved,

3 chronic resolved, and 6 HBV-free healthy controls) expression of target genes (CX3CL1, CX3CR1, TGF-β1) and metabolic pathways using hallmark pathways (hallmark_glycolysis, hallmark_OXPHOS, hallmark_FA metabolism, hallmark_PI3K AKT MTOR). For each cell subset, four principal component analyses were performed on gene expression in the Glycolysis (153 genes), OXPHOS (182 genes), FA metabolism (128 genes) and PI3K AKT MTOR (90 genes) pathways.

Statistical analysis

Statistical analyses were performed using GraphPad Prism 9 software (CA, USA), using two-way ANOVA with Dunnett's or Šídák's multiple comparison tests, non-parametric Kruskal-Wallis test, Friedman test with Dunn's multiple comparisons tests, and Wilcoxon matched-pairs signed rank test. Significance threshold was placed at p-value <0.05.

Supplementary figure legends

Supplementary figure 1. Gating strategy to depict cDC1s, cDC2s and pDCs by multiparametric flow cytometry

(**a**) Debris were excluded based on forward scatter (FCS) and side scatter (SSC) parameters. Doublets and dead cells were excluded to select $CD45^+$ cells among living cells. Pan-DCs were identified within $CD45^+$ cells as $Lin^-HLA\text{-}DR^+$. Among Pan-DCs, cDC1s were defined as $CD11c^{dim}$ $BDCA3^+$, cDC2s as $CD11c^{bright}$ $BDCA1^+$ and pDCs as $CD11c^-$ $BDCA2^+$. Representative flow cytometry plots from one donor. (**b**) Dot plots illustrating CD80, CD40 and CD86 expression by cDC2s, pDCs and cDC1s.

Supplementary figure 2. HBV slightly influenced the expression level of activation molecules and TRAIL on DCs in response to TLR stimulation.

PanDCs purified from HDs were cultured in the presence or not of HBV (MOI 100, 250, 1000) for 4h, and TLR3/7/8/9-L were then added for 16h. The subsequent modulation of activation markers (CD40, CD80, CD86) and TRAIL on DC subsets was measured by flow cytometry. (**a-c**) Comparative expression levels (MFI) of CD40, CD80, CD86 and TRAIL on CD40-, CD80-, CD86- and TRAIL-expressing cDC1s (**a**), cDC2s (**b**) and pDCs (**c**) after culture without (-) or with HBV at different MOIs and upon TLR stimulation. Green colored background indicate TLR-L directly activating the DC subset expressing the corresponding TLR. Horizontal lines indicate means. P values were calculated using two-way ANOVA with Dunnett's multiple comparison tests. * $p < 0.05$, ** $p < 0.01$. n = 9-10 independent experiments.

Supplementary figure 3. HBV and inactivated HBV-UV affect similarly the expression level of activation molecules and TRAIL on DCs in response to TLR stimulation.

PanDCs purified from HDs were cultured in the presence of inactivated UV-HBV or HBV (MOI 1000) for 4h, and a mixture of TLR3/7/8/9-L was then added for 16h. The subsequent modulation of activation markers (CD40, CD80, CD86) and TRAIL on DC subsets were measured by flow cytometry. (**a-c**) The frequency of CD40-, CD80-, CD86- and TRAIL-expressing cDC1s (**a**), cDC2s (**b**) and pDCs (**c**) after culture without (-) or with TLR stimulation (+TLR stim). n = 5 independent experiments.

Supplementary figure 4. HBsAg disturbed the expression level of activation molecules and TRAIL on DCs.

PanDCs purified from HDs were cultured in the presence or not of HBsAg for 4h, and TLR3/7/8/9-L were then added for 16h. The subsequent modulation of activation markers (CD40, CD80, CD86) and TRAIL on DC subsets was measured by flow cytometry. (**a-c**) Comparative expression levels (MFI) of CD40, CD80, CD86 and TRAIL on CD40-, CD80-, CD86- and TRAIL-expressing cDC1s (**a**), cDC2s (**b**) and pDCs (**c**) after culture without (-) or with HBsAg and upon TLR stimulation. Orange colored background indicates TLR-L directly activating the DC subset expressing the corresponding TLR. Horizontal lines indicates means. P values were calculated using two-way ANOVA with Šídák's multiple comparison tests. * $p < 0.05$, ** $p < 0.01$, *** $p < 0.001$, **** $p < 0.0001$. n = 9-10 independent experiments.

Supplementary figure 5. Dose-dependent effect of HBsAg on the expression level of activation molecules on DCs.

PanDCs purified from HDs were cultured in the presence of HBsAg (1µg/ml, 5µg/ml, 25µg/ml). The subsequent modulation of activation markers (CD40, CD80, CD86) on DC subsets was measured by flow cytometry. (**a-c**) Frequencies of CD40-, CD80- and CD86--expressing cDC1s (**a**), cDC2s (**b**) and pDCs (**c**). n = 2 independent experiments.

Supplementary figure 6. Effect of TLR and CLR pathway inhibition on the expression of activation molecules and TRAIL by HBV-exposed DCs

PanDCs purified from HDs were pre-treated for one hour with inhibitors of the TLR- and CLR-mediated pathways amlexanox (Amx) and R406 respectively before adding HBV (MOI 1000) for 4h. A mixture of TLR3/7/8/9-L was then added for 16h. The subsequent modulation of activation markers (CD40, CD80, CD86) and TRAIL on DC subsets were measured by flow cytometry. (**a-c**) The frequency of CD40-, CD80-, CD86- and TRAIL-expressing cDC1s (**a**), cDC2s (**b**) and pDCs (**c**) after culture without (-) or with TLR stimulation (+TLR stim). P values were calculated using the Kruskal-Wallis test (non-parametric) for multiple comparison tests. * $p < 0.05$, ** $p < 0.01$. n = 5 independent experiments.

Supplementary figure 7. Effect of TLR and CLR pathway inhibition on the expression of activation molecules and TRAIL by HBsAg-exposed DCs

PanDCs purified from HDs were pre-treated for one hour with inhibitors of the TLR- and CLR-mediated pathways amlexanox (Amx) and R406 respectively before adding HBsAg (25µg/ml) for 4h. A mixture of TLR3/7/8/9-L was then added for 16h. The subsequent modulation of activation markers (CD40, CD80, CD86) and TRAIL on DC subsets were measured by flow cytometry. (**a-c**) The frequency of CD40-, CD80-, CD86- and TRAIL-expressing cDC1s (**a**), cDC2s (**b**) and pDCs (**c**) after culture without (-) or with TLR stimulation (+TLR stim). P values were calculated using the Kruskal-Wallis test (non-parametric) for multiple comparison tests. * $p < 0.05$, ** $p < 0.01$. n = 5 independent experiments.

Supplementary figure 8. Secretion of cytokines and chemokines by DCs in response to TLR stimulation that were not affected by HBV

PanDCs purified from HDs were cultured in the presence or not of HBV (MOI 1000) for 4h, and TLR3/7/8/9-L were then added for 16h. Secretion of cytokines/chemokines was measured in culture supernatants by Luminex. (**a-c**) Comparative concentration of cytokines and chemokines produced by DCs upon TLR stimulation after culture with or without HBV and involved in inflammation (**a**), immunity (**b**) and tolerance (**c**). Horizontal lines indicate means. P values were calculated using two-way ANOVA with Šídák's multiple comparisons tests. n = 10 independent experiments.

Supplementary figure 9. CX3CL1 production by DCs was induced by HBV in a dose-dependent but TLR-independent manner.

PanDCs purified from HDs or medium alone were cultured in the presence or not of HBsAg or HBV (MOI 100, 250 or 1000) for 4h, and polyI:C was then added for 16h. Secretion of fracktalkine was measured in culture supernatants by Luminex. (Left part) Concentration of CX3CL1 after culture medium without DCs was harvested after culture with or without HBV or HBsAg (n = 2). (Right part) Concentration of CX3CL1 produced by non-stimulated or PolyIC-stimulated DCs after culture with increasing concentrations of HBV (n = 3). Results are shown as mean + SEM.

Supplementary figure 10. Secretion of cytokines and chemokines by DCs in response to TLR stimulation that were not affected by HBsAg

PanDCs purified from HDs were cultured in the presence or not of HBsAg for 4h, and TLR3/7/8/9-L were then added for 16h. Secretion of cytokines/chemokines was measured in culture supernatants by Luminex. (**a-c**) Comparative concentration of cytokines and chemokines produced by DCs upon TLR stimulation after culture with or without HBsAg and involved in inflammation (**a**), immunity (**b**) and tolerance (**c**). Horizontal lines indicate means.

P values were calculated using two-way ANOVA with Šídák's multiple comparisons tests. n = 10 independent experiments.

Supplementary figure 11. RNA sequencing analysis of HBsAg induced modulations of DCs

PanDCs were first enriched from PBMC derived from HDs using the EasySep PanDC pre-enrichment Kit and then FACS-sorted. Purified sorted PanDCs were cultured in the presence or not of HBsAg for 16h and RNA sequencing analysis were performed. (**a**) Heatmap showing log (value+1) relative expression level of top differentially expressed genes in DCs cultured with HBsAg (PanDCs_HBsAg) or not (PanDC_Control). (**b**) Comparative expression of CXCL8, CCL22, CCL2 between control and HBsAg-exposed PanDCs. P-values were calculated using Wilcoxon paired test. * $p < 0.05$. n = 4 independent experiments.

Supplementary figure 12. Energetic metabolic profile of DCs exposed to recombinant human fractalkine and TGF-β

PanDCs purified from HDs were cultured in the presence or not of recombinant human (rh) fractalkine, rh TGF-β or the mixture of both (Mix) for 18h at 37°C, 5% CO_2, then incubated with the different inhibitors and puromycin following the SCENITH procedure. Translation levels were analysed by measuring the MFI of puromycin within each subset using flow cytometry and metabolic profiles were calculated. (**a**) Translation levels within cDC1s, cDC2s and pDCs. P-values were calculated using Friedman test (non-parametric) with Dunn's multiple comparisons tests. * $p < 0.05$. (**b**) Metabolic profiles of cDC1s, cDC2s and pDCs. P-values were calculated using two-way ANOVA test with Dunnett's multiple comparisons tests. Plots show mean with standard deviation. n = 5 independent experiments. FAO: fatty acids oxidation; AAO: amino acids oxidation.

Supplementary figure 13. CX3CR1 and TGF-β expression by intrahepatic CD45+ cells of patients with different disease progression

(**a-b**) Expression of CX3CR1 (**a**) and TGF-β1 (**b**) transcripts within intrahepatic CD45+ cells of patients according to disease stage (exploitation of scRNAseq public dataset GSE182159 from Zhang *et al.*[4]). HC, healthy control; AR, acute recovery; IA, immune activation; IT, immune tolerant; CR chronic resolved.

References:

1. Faure-Dupuy S, Delphin M, Aillot L, et al. Hepatitis B virus-induced modulation of liver macrophage function promotes hepatocyte infection. *Journal of hepatology*. Dec 2019;71(6):1086-1098. doi:10.1016/j.jhep.2019.06.032
2. Aillot L, Bonnin M, Ait-Goughoulte M, et al. Interaction between Toll-Like Receptor 9-CpG Oligodeoxynucleotides and Hepatitis B Virus Virions Leads to Entry Inhibition in Hepatocytes and Reduction of Alpha Interferon Production by Plasmacytoid Dendritic Cells. *Antimicrobial agents and chemotherapy*. Apr 2018;62(4)doi:10.1128/aac.01741-17
3. Delphin M, Faure-Dupuy S, Isorce N, et al. Inhibitory Effect of IL-1β on HBV and HDV Replication and HBs Antigen-Dependent Modulation of Its Secretion by Macrophages. *Viruses*. Dec 30 2021;14(1)doi:10.3390/v14010065
4. Zhang C, Li J, Cheng Y, et al. Single-cell RNA sequencing reveals intrahepatic and peripheral immune characteristics related to disease phases in HBV-infected patients. *Gut*. 2023;72(1):153. doi:10.1136/gutjnl-2021-325915

Supplementary Table 1

Group	Acute hepatitis n = 5	Immunotolerant n = 8	HBe+ immune active n = 7	HBe- immune active n = 10	Inactive carrier n = 19	Treated patients n = 21
**Sex (M/F)**	5/0	5/3	7/0	9/1	12/7	14/7
**Age, median (IQR)**	30 (26 – 52)	19.5 (18 – 23.25)	27 (19 – 39)	23.5 (19.75 – 34.75)	43 (32 – 49)	43 (33 – 58.5)
**HBeAg, n (%)**						
**Negative**	3 (60%)	0 (0%)	0 (0%)	10 (100%)	19 (100%)	17 (81%)
**Positive**	2 (40%)	8 (100%)	7 (100%)	0 (0%)	0 (0%)	4 (19%)
**HBsAg (UI/ml), median (IQR)**	1000 (0.75 – 1000*)	126713 (99055 – 149850)	25486 (14919 – 127400)	8421 (975.2 – 14868)	431 (20.7 – 9014)	2838 (536.3 – 13442)
**HBV DNA (log_{10} UI/ml), median (IQR)**	3.48 (3.06 – 6.59)	8.52 (8.3 – 8.84)	7.93 (6.53 – 9)	6.35 (4.23 – 8.01)	1.71 (1.26 – 2.61)	1 (1-1)
**ALT (UI/l), median (IQR)**	2820 (766 – 3756)	26 (15 – 44.3)	69 (49 – 352)	75 (45– 177)	38 (25 – 45)	26 (22.5 – 32)
**AST (UI/l), median (IQR)**	322 (244 – 2479)	24 (20.3 – 35.5)	37 (18 – 206)	47 (31 – 94)	27 (19 – 40)	27 (16 – 34)
**Antiviral treatment**						
**Entecavir**	0	0	0	0	0	9
**Tenofovir**	0	0	0	0	0	11
**Other**	0	0	0	0	0	1

* Exact values not provided for 3 patients (positive result >1000UI/ml)

Supplementary Figure 1

(**a**)

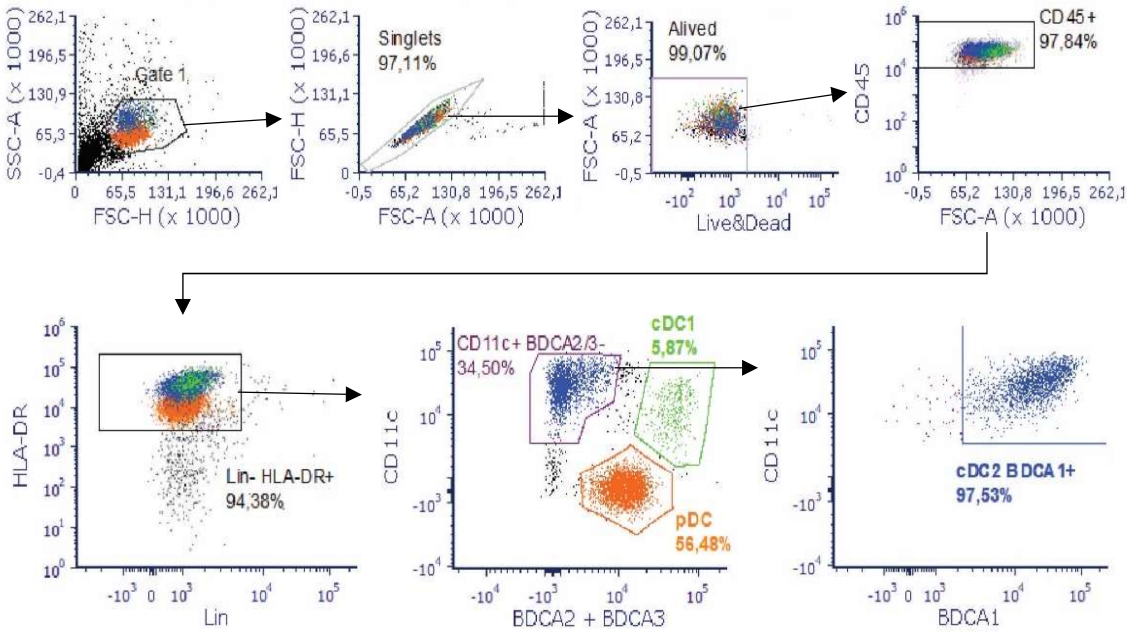

(**b**)

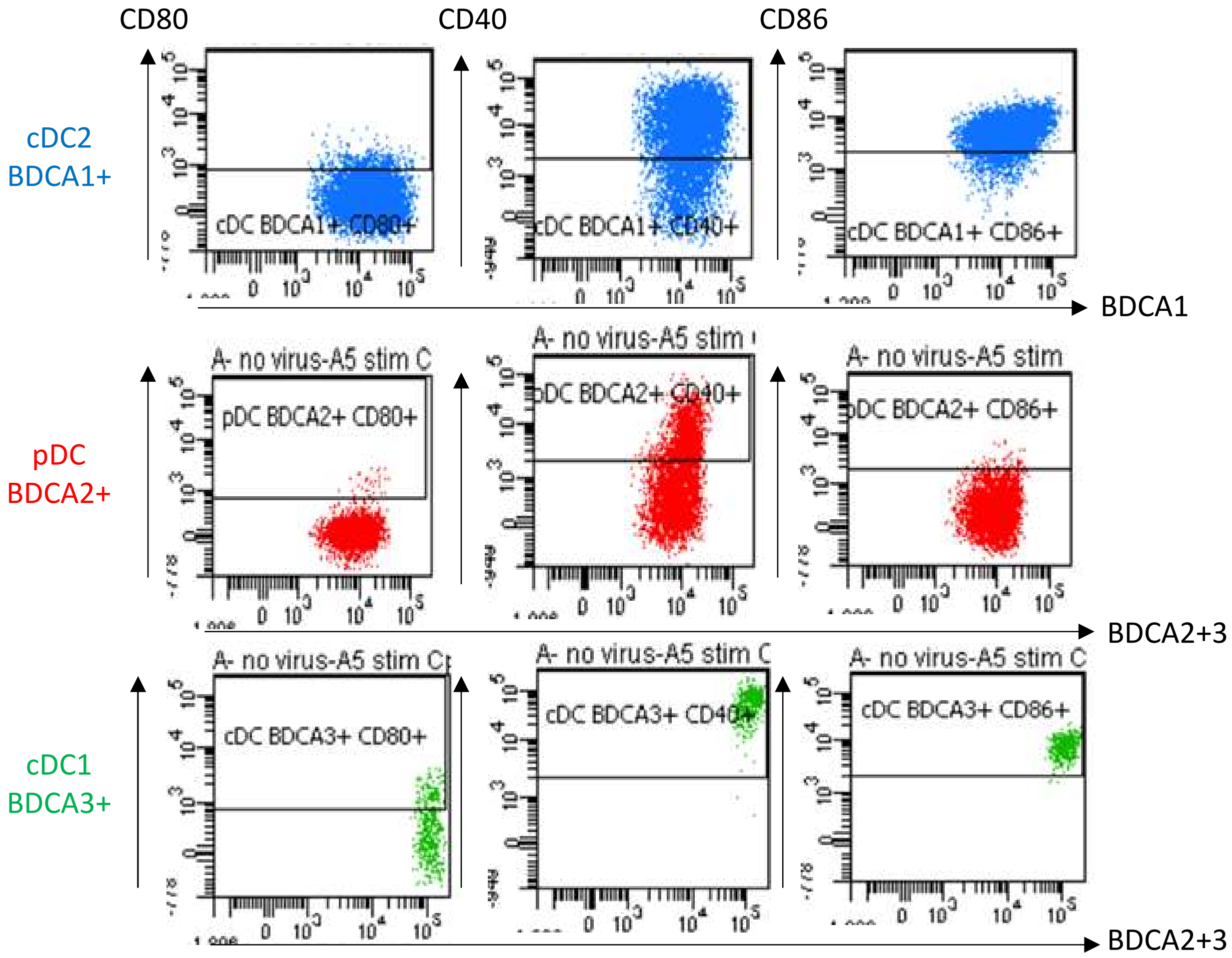

Supplementary Figure 2

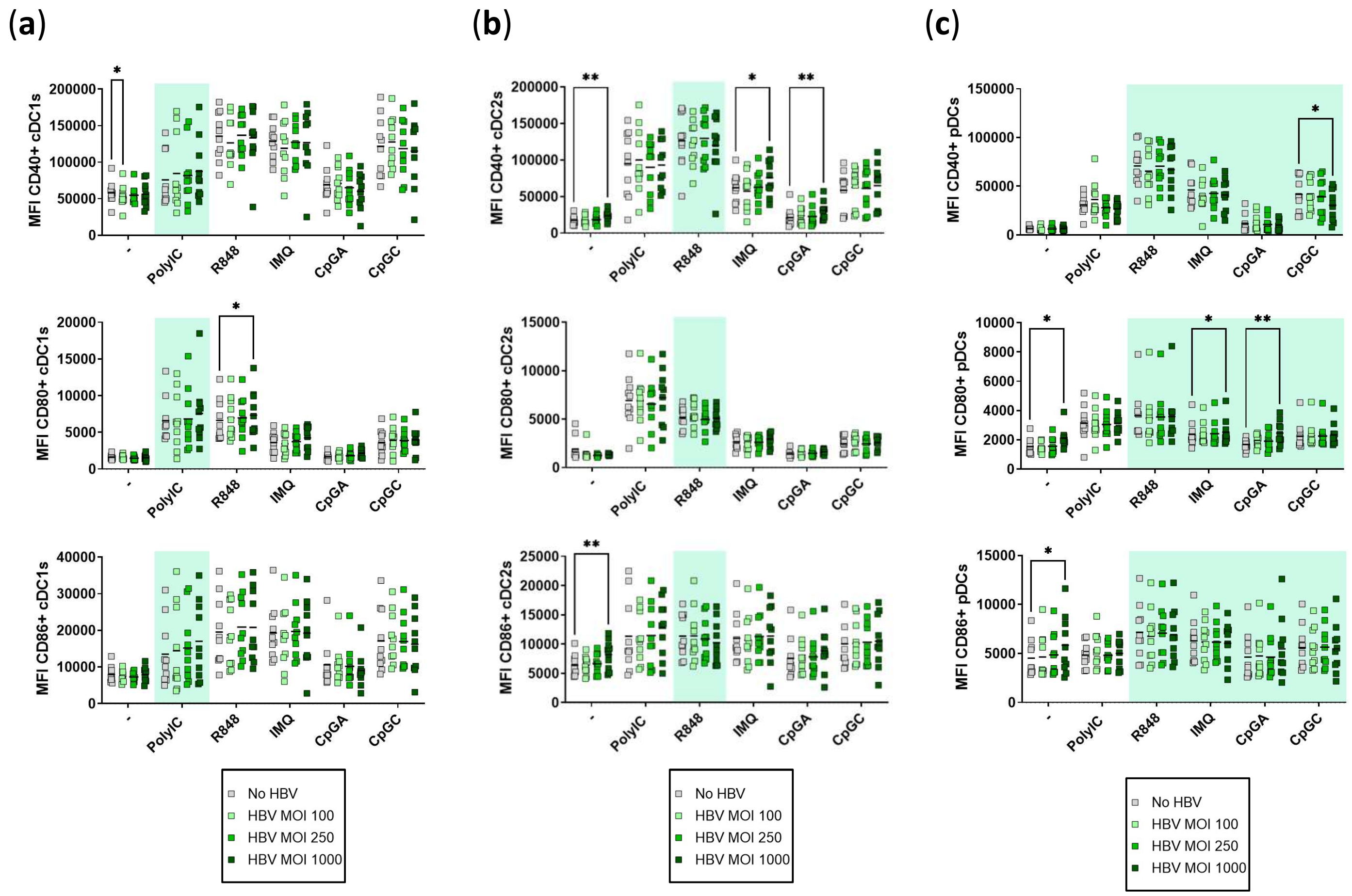

Supplementary Figure 3

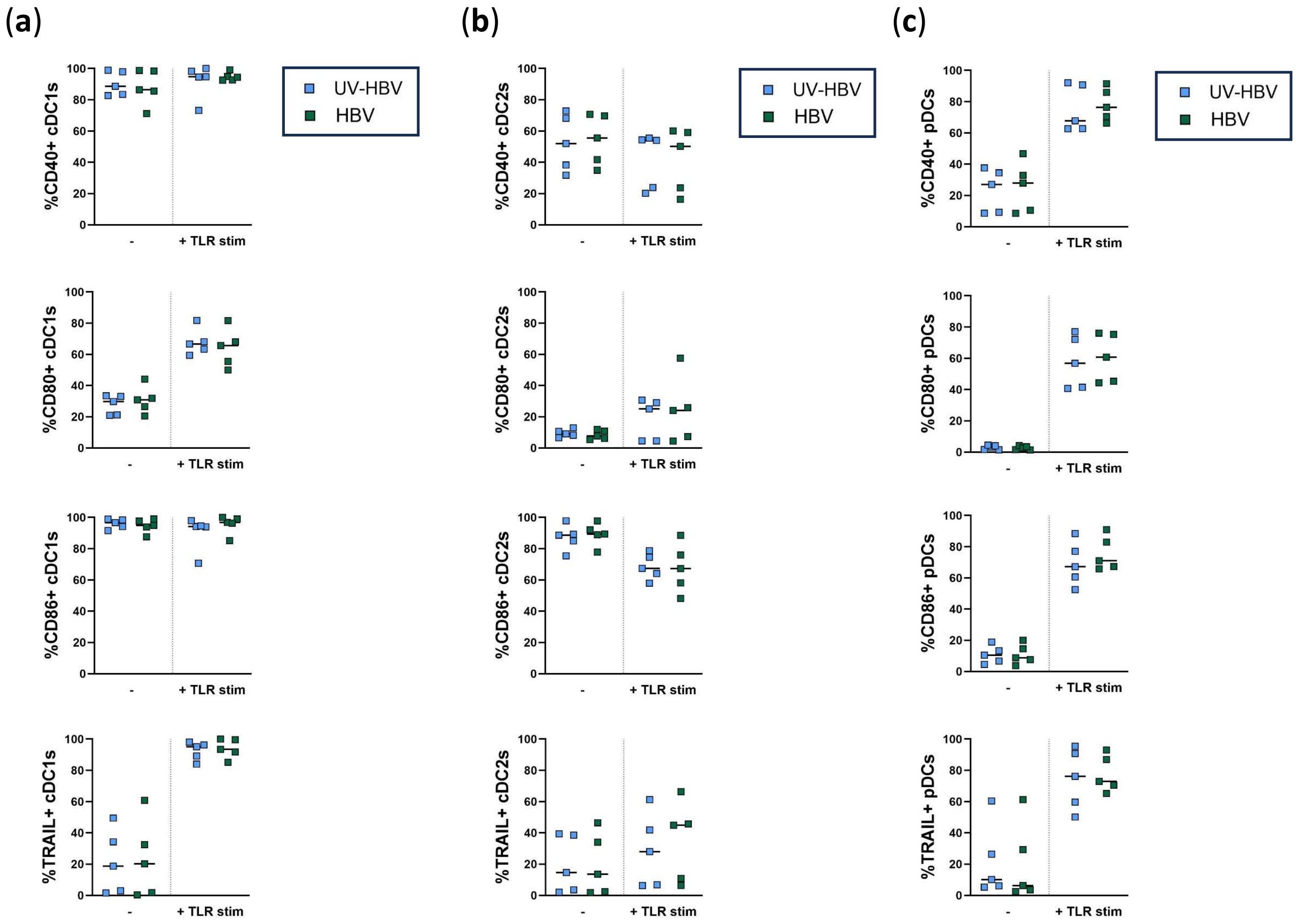

Supplementary Figure 4

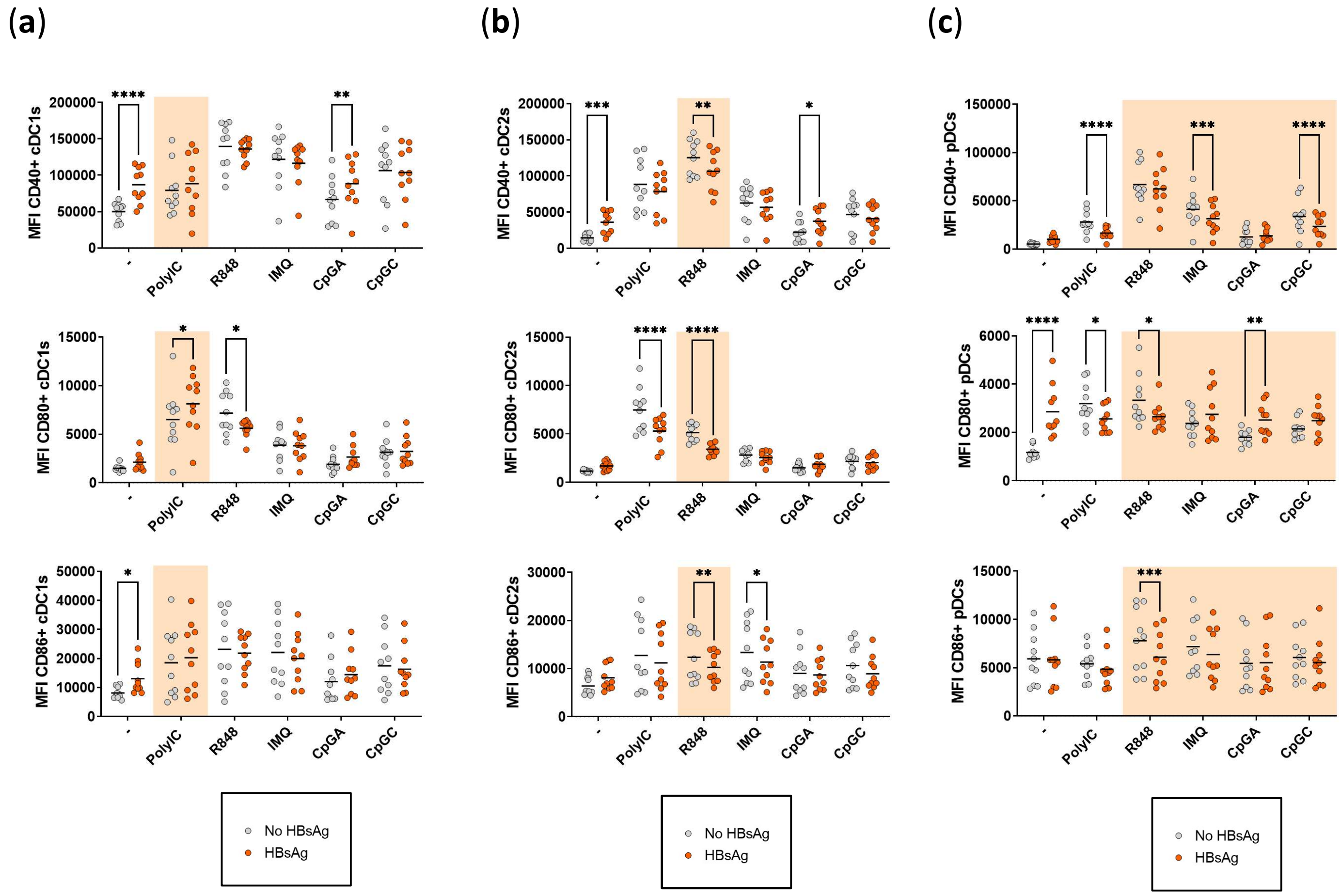

Supplementary Figure 5

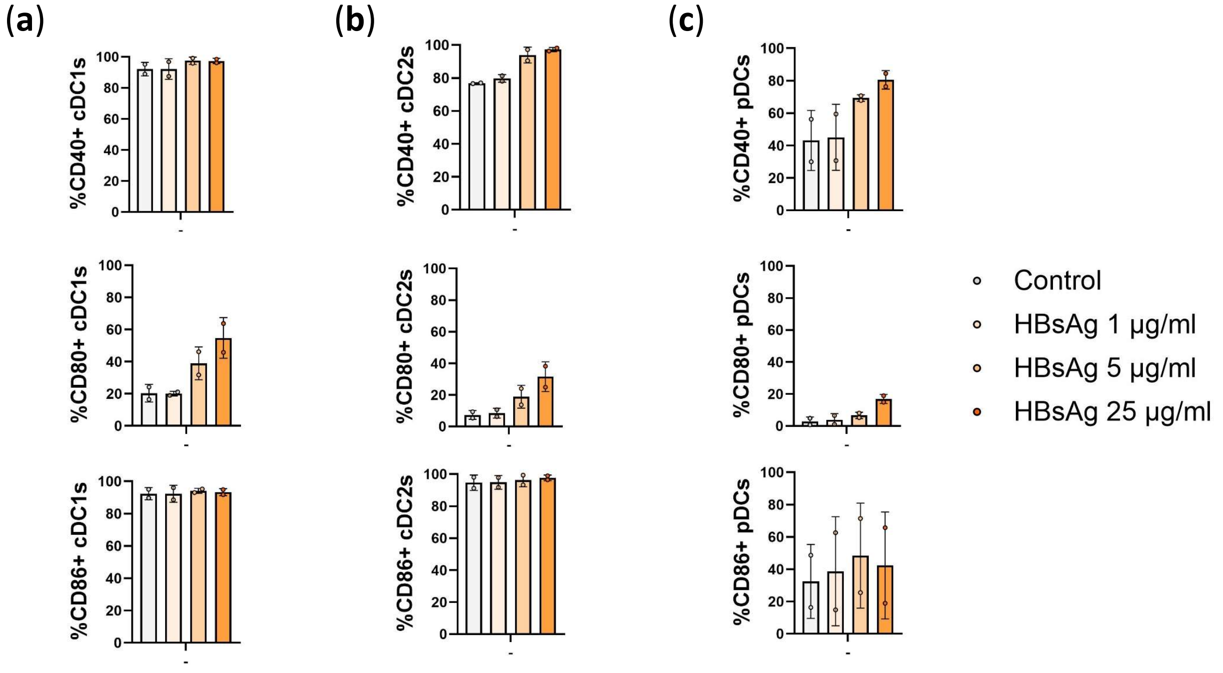

Supplementary Figure 6

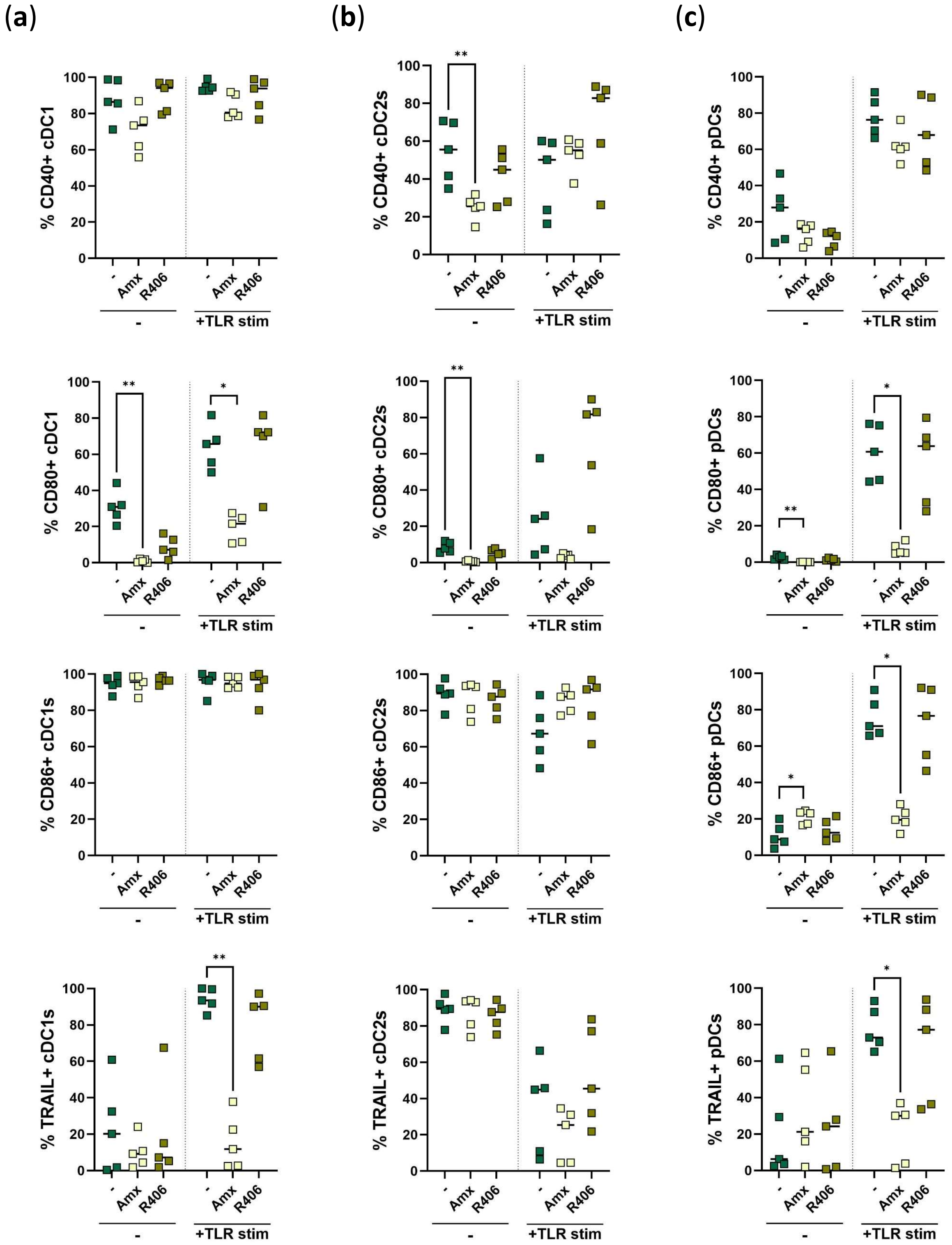

Supplementary Figure 7

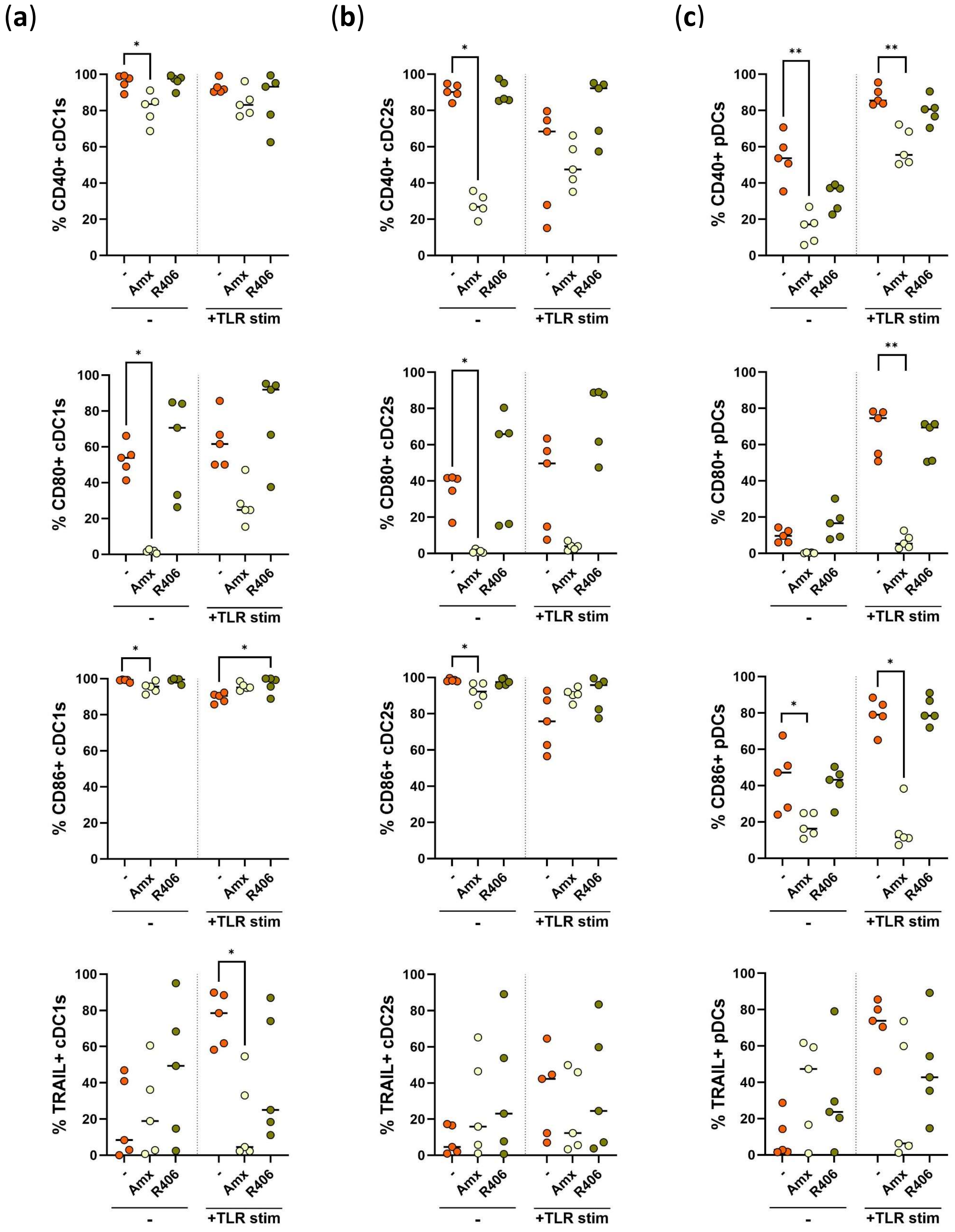

Supplementary Figure 8

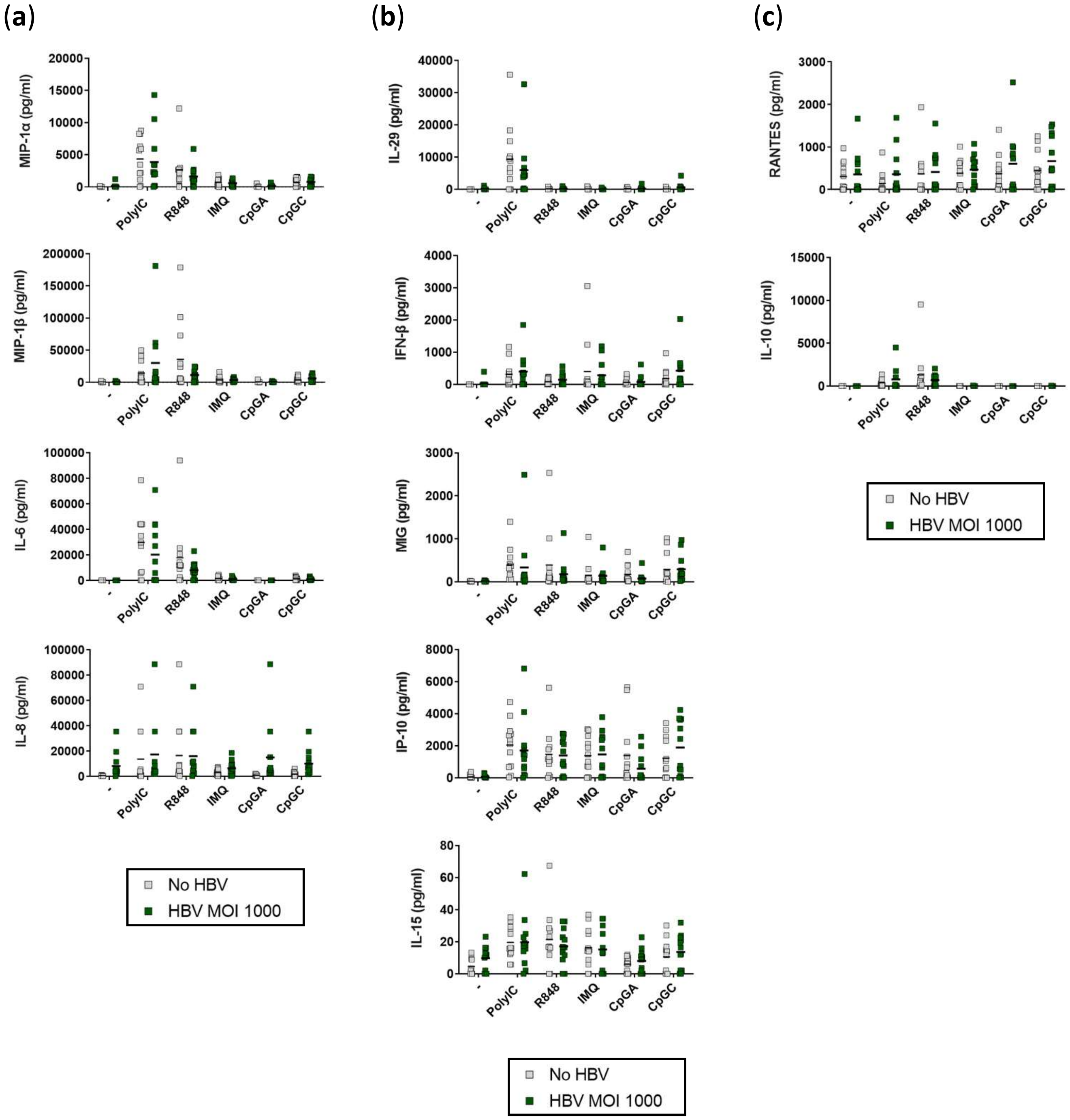

Supplementary Figure 9

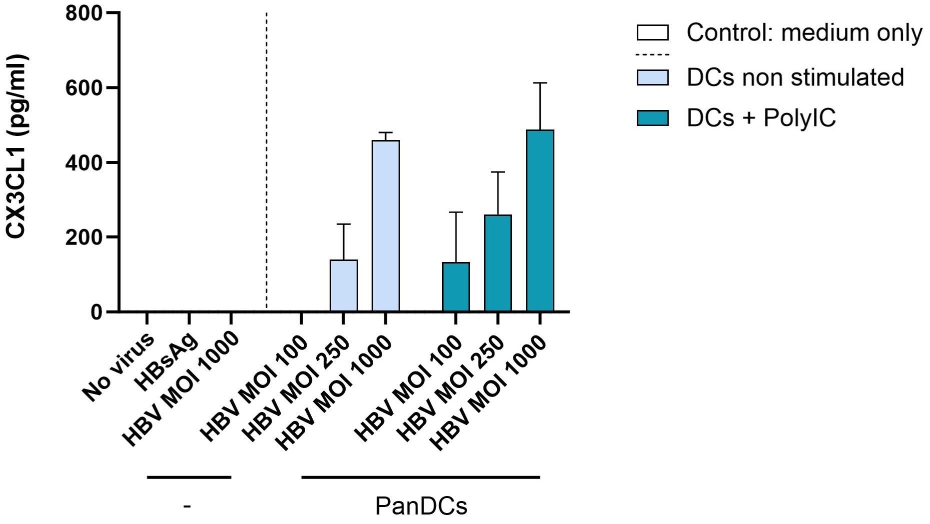

Supplementary Figure 10

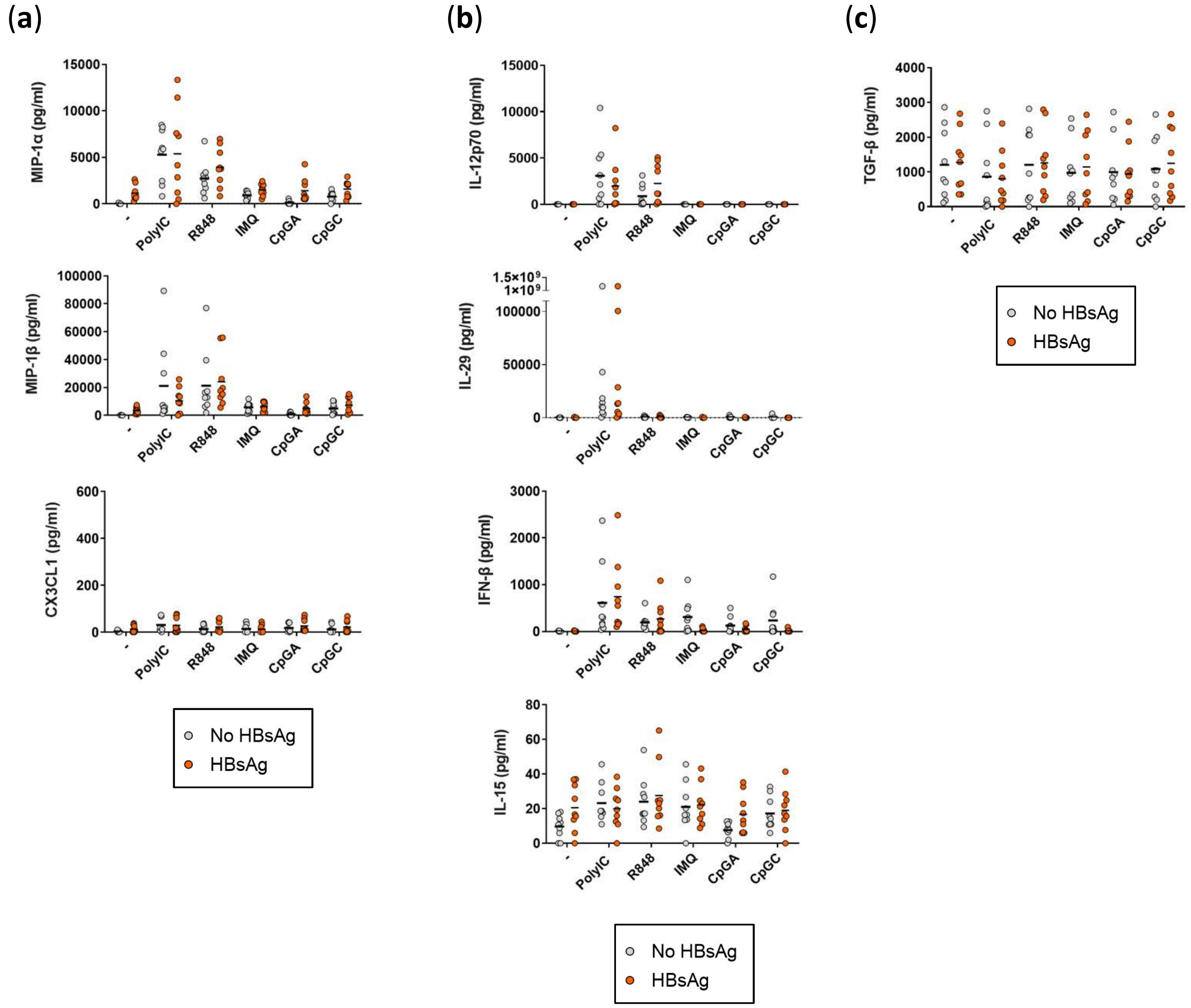

Supplementary Figure 11

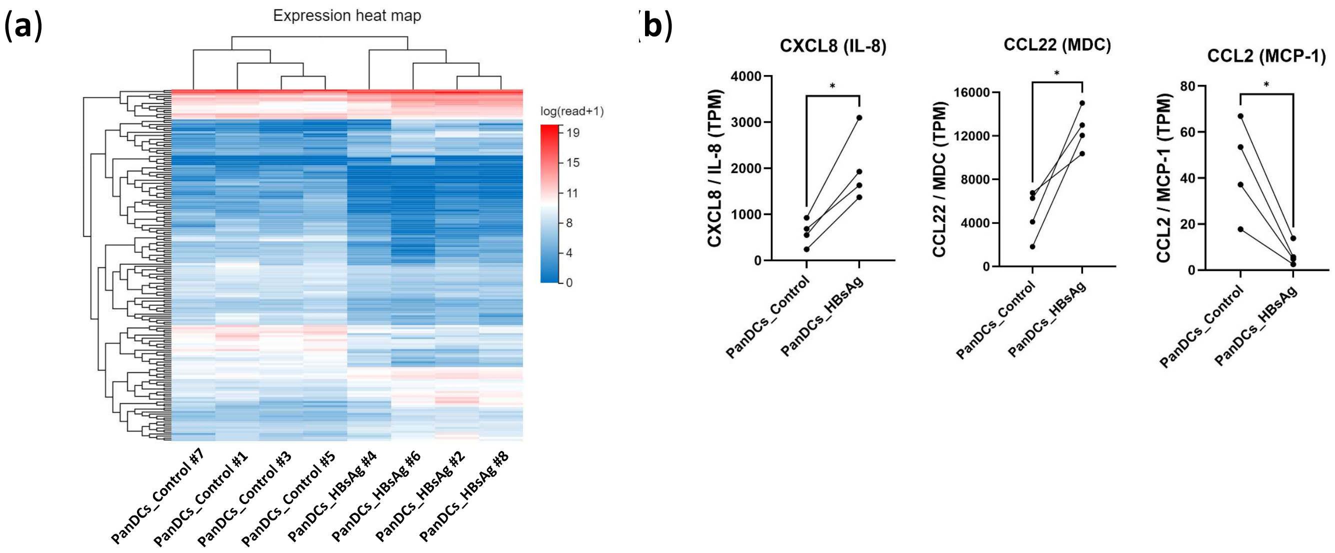

Supplementary Figure 12

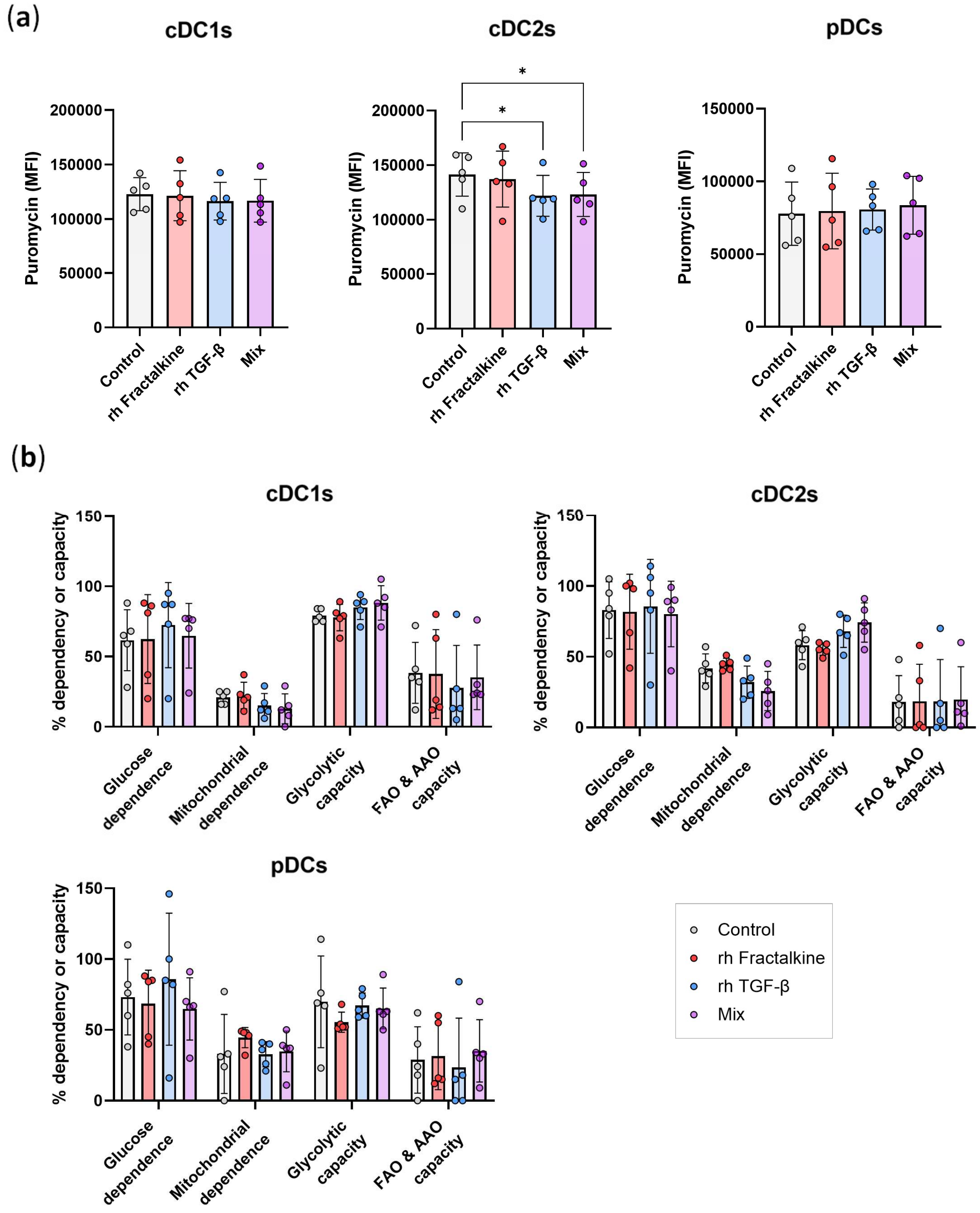

Supplementary Figure 13

(a)

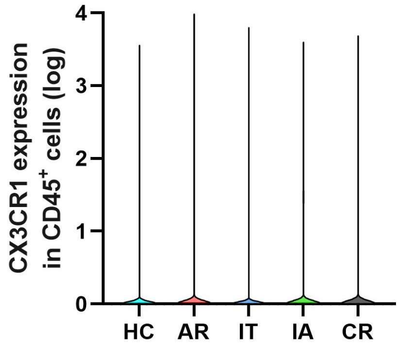

(b)

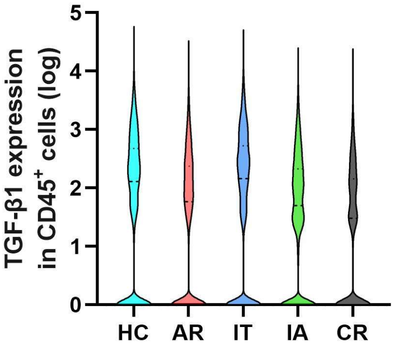

Supplement: Supplementary file 1 [file hc9-9-e0625-s001.pdf]
